# Plasma‐mediated enhancement of enzyme secretion in *Aspergillus oryzae*

**DOI:** 10.1111/1751-7915.13696

**Published:** 2020-11-05

**Authors:** Mayura Veerana, Sarmistha Mitra, Se‐Hoon Ki, Soo‐Min Kim, Eun‐Ha Choi, Taek Lee, Gyungsoon Park

**Affiliations:** ^1^ Department of Plasma Bioscience and Display Kwangwoon University Seoul 01897 Korea; ^2^ Department of Electrical and Biological Physics Kwangwoon University Seoul 01897 Korea; ^3^ Department of Chemical Engineering Kwangwoon University Seoul 01897 Korea

## Abstract

Technical bottlenecks in protein production and secretion often limit the efficient and robust industrial use of microbial enzymes. The potential of non‐thermal atmospheric pressure plasma to overcome these technical barriers was examined. Spores of the fermenting fungus *Aspergillus oryzae* (*A. oryzae*) were submerged in potato dextrose broth (PDB) (5 × 10^6^ per ml) and treated with micro dielectric barrier discharge plasma at an input voltage of 1.2 kV and current of 50 to 63 mA using nitrogen as the feed gas. The specific activity of α‐amylase in the broth was increased by 7.4 to 9.3% after 24 and 48 h of plasma treatment. Long‐lived species, such as NO_2_^−^ and NO_3_^−^, generated in PDB after plasma treatment may have contributed to the elevated secretion of α‐amylase. Observations after 24 h of plasma treatment also included increased accumulation of vesicles at the hyphal tip, hyphal membrane depolarization and higher intracellular Ca^2+^ levels. These results suggest that long‐lived nitrogen species generated in PDB after plasma treatment can enhance the secretion of α‐amylase from fungal hyphae by depolarizing the cell membrane and activating Ca^2+^ influx into hyphal cells, eventually leading to the accumulation of secretory vesicles near the hyphal tips.

## Graphical abstract

The effect of plasma on expression and extracellular secretion of α‐amylase and the underlying mechanisms were investigated using *Aspergillus oryzae*, a fermenting filamentous fungus. Our data suggest that plasma can enhance the secretion of α‐amylase from fungal hyphae by depolarizing the cell membrane and activating Ca^2+^ influx into hyphal cells, eventually leading to the accumulation of secretory vesicles near the hyphal tips.

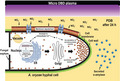

## Introduction

Compared to chemical catalysts, the industrial, medicinal and environmental conservation uses of enzymes produced by living organisms have received much attention because of their efficiency and safety. The global enzyme market was estimated as $10 billion in 2019 and is predicted to increase to $14.7 billion by 2025. Thus, the economic value of biocatalyst enzymes is growing (2020).

Biologically active enzymes can be obtained from plants, animals and microorganisms (Sundarram and Murthy, 2014). Compared to enzymes from plants and animals, microbial enzymes are preferred in industrial applications because of their stability and performance under various physical and chemical conditions (Singh *et al*., 2016; Raveendran *et al*., 2018). Enzymes produced by microorganisms are frequently used in industrial and medical applications that include pharmaceutical therapy, diagnostics, food processing, degradation of animal feed, synthesis of biodegradable polymers, recycling of paper and pulp, environment‐friendly leather and textile processing, cosmetics, detergents, organic synthesis and waste management (Singh *et al*., 2016).

Since microorganisms can grow faster in smaller volumes than plants and animals, microbial enzyme production is cost‐effective and easily controlled (Gurung *et al*., 2013). However, there are still several technical barriers to the industrial‐scale production. Enzyme proteins in natural microbial sources are typically produced at very low concentrations or as part of a complex mixture. In particular, heterologous enzymes (non‐host gene product) are usually produced much less efficiently than native proteins (Tsuchiya *et al*., 1994; Nakajima *et al*., 2006; Ito *et al*., 2007; Jin *et al*., 2007). In addition, several steps in protein secretion, such as translation, translocation, folding, transport and secretion, can be potential bottlenecks for industrial enzyme production (Tsuchiya *et al*., 1992). Several genetic engineering technologies have been proposed and applied to improve the expression and extracellular secretion of microbial enzymes (Idiris *et al*., 2010; Mahalik *et al*., 2014). However, concerns over the safety of genetically modified organisms have hindered their widespread use. Alternate technologies need to be developed to complement this technical drawback.

Non‐thermal atmospheric pressure plasma (NTAPP) has been proposed as an alternative to improve the efficiency of microbial enzyme production. Over the last decade, several studies have demonstrated various applications of NTAPP in medicine, agriculture, decontamination and food bioprocessing (Weltmann and von Woedtke, 2016; Hati *et al*., 2018; Ito *et al*., 2018; Mandal *et al*., 2018). NTAPP is generated when high energy is applied to a gas. The mixture of excited and charged gas species that is produced includes reactive oxygen and nitrogen species (RONS), such as superoxide (O_2_^‐^), ozone (O_3_), hydroxyl (OH), hydroperoxyl (HO_2_), nitric oxide (NO), nitrogen dioxide (NO_2_) and dinitrogen pentoxide (N_2_O_5_) (Sakiyama *et al*., 2012; Machala *et al*., 2013). Since RONS are involved in the activation and inactivation of microbial cell development, NTAPP can have similar effects on microbial cells depending on the dose applied (Scott and Eaton, 2008; Lambeth and Neish, 2014). NTAPP‐induced inactivation or decontamination of microorganisms has been demonstrated in many studies (for review see Gilmore *et al*., 2018; Mandal *et al*., 2018; Siddique *et al*., 2018). However, only a few reports have described the activation of microbial cell vitality and enzyme production by NTAPP (Farasat *et al*., 2018; Veerana *et al*., 2019; Gao *et al*., 2020).

Optimization of the viability and cellular function of beneficial microorganisms is essential for efficient industrial and medicinal applications. Presently, we investigated the potential of NTAPP to enhance microbial metabolism by assessing the expression and secretion of α‐amylase enzyme using *Aspergillus oryzae* as the model microorganism. *A. oryzae* is a filamentous fungus that is widely used in the food fermentation industry because of its ability to secrete a variety of high‐value industrial enzymes, such as α‐amylase, protease, pectinase and β‐galactosidase (Rahardjo *et al*., 2005; Meneghel *et al*., 2014; Carevic *et al*., 2015; Kitamoto, 2015). Filamentous fungi are considered one of the most versatile hosts with the ability to produce many eukaryotic proteins. This reflects the ability of these fungi to produce many native and heterologous proteins, and their efficient secretory systems, compared to other model microorganisms that include the yeast *Saccharomyces cerevisiae* and the bacterium *Escherichia coli* (Nevalainen *et al*., 2005). α‐Amylase hydrolyses the internal α‐1, 4‐glycosidic linkages in starch to yield glucose and maltose (Adewale and Oladejo, 2009). α‐Amylase occupies approximately 25% to 33% of the world enzyme market, and its’ demand for industrial activities that include chemistry, food, textile, pharmaceutical and others is increasing (Nguyen *et al*., 2002). Evaluating the effects of NTAPP on the expression and extracellular secretion of α‐amylase in *A. oryzae* could provide valuable information for technological innovation in the fermentation industry.

## Results

### N_2_ plasma enhances α‐amylase secretion in *A. oryzae*

**Fig. 1:**
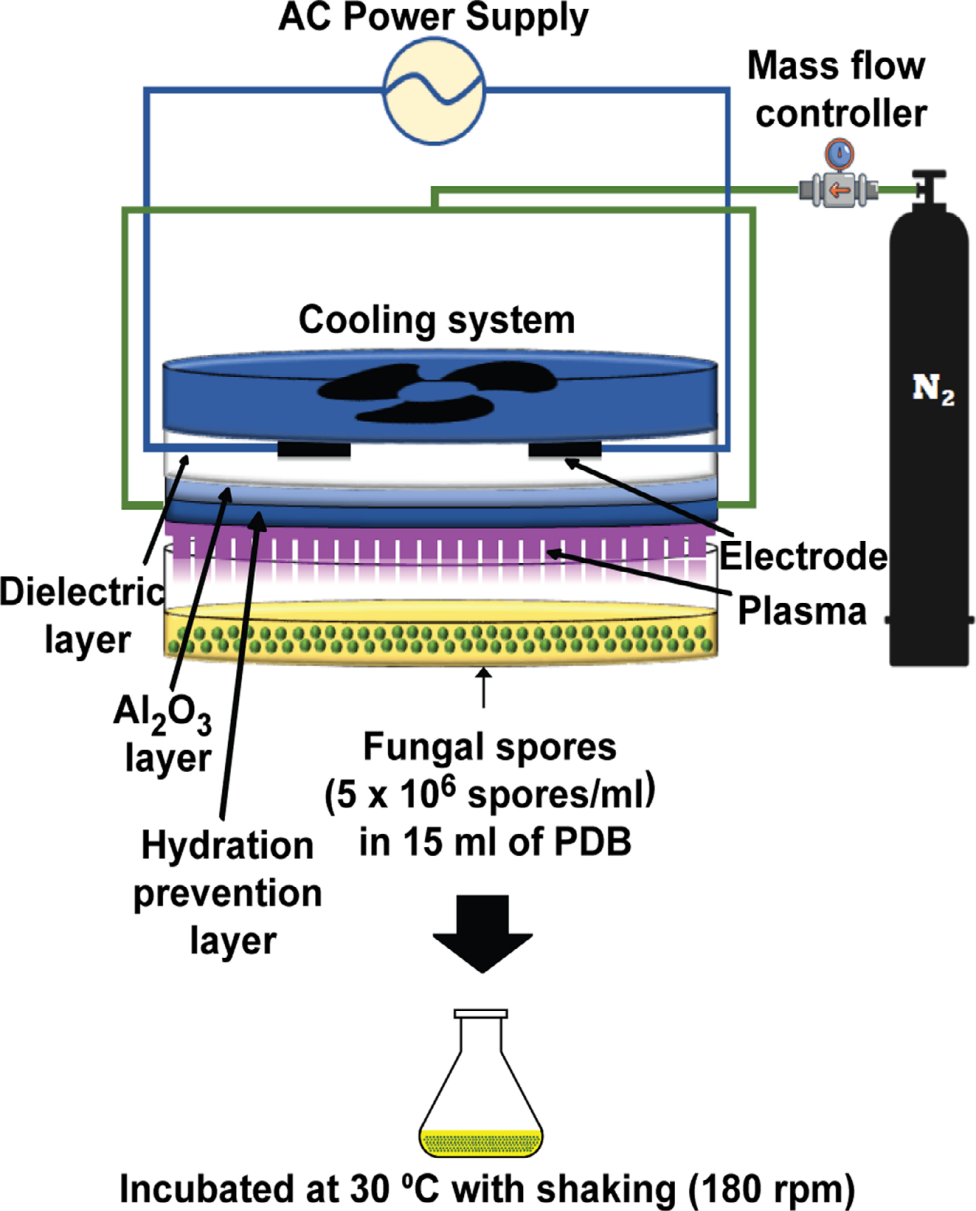
Treatment of fungal spores with microDBD plasma. Schematic view of the micro DBD plasma device and experimental set‐up for treatment of fungal spores submerged in PDB.

Expression and secretion of α‐amylase in *A. oryzae* were analysed by measuring the intracellular and extracellular levels of α‐amylase protein 24, 48 and 72 h after plasma treatment of fungal spores. This time range was selected because the extracellular α‐amylase activity of *A. oryzae* in PDB solution (with no treatment) was high during 24–120 h of incubation (Fig. S1). *A. oryzae* spores (5 × 10^6^ spores ml^−1^) in 15 mL PDB solution were treated with either N_2_ gas (control) or plasma for 5 min (Fig. 1). We previously observed that germination of *A. oryzae* spores was most efficiently activated when treated in PDB with the same micro dielectric barrier discharge (DBD) nitrogen plasma (same discharge condition) for 5 min (Veerana *et al*., 2019). Therefore, in the present study samples were treated with plasma for 5 min as a potential activating plasma dose.

**Fig. 2:**
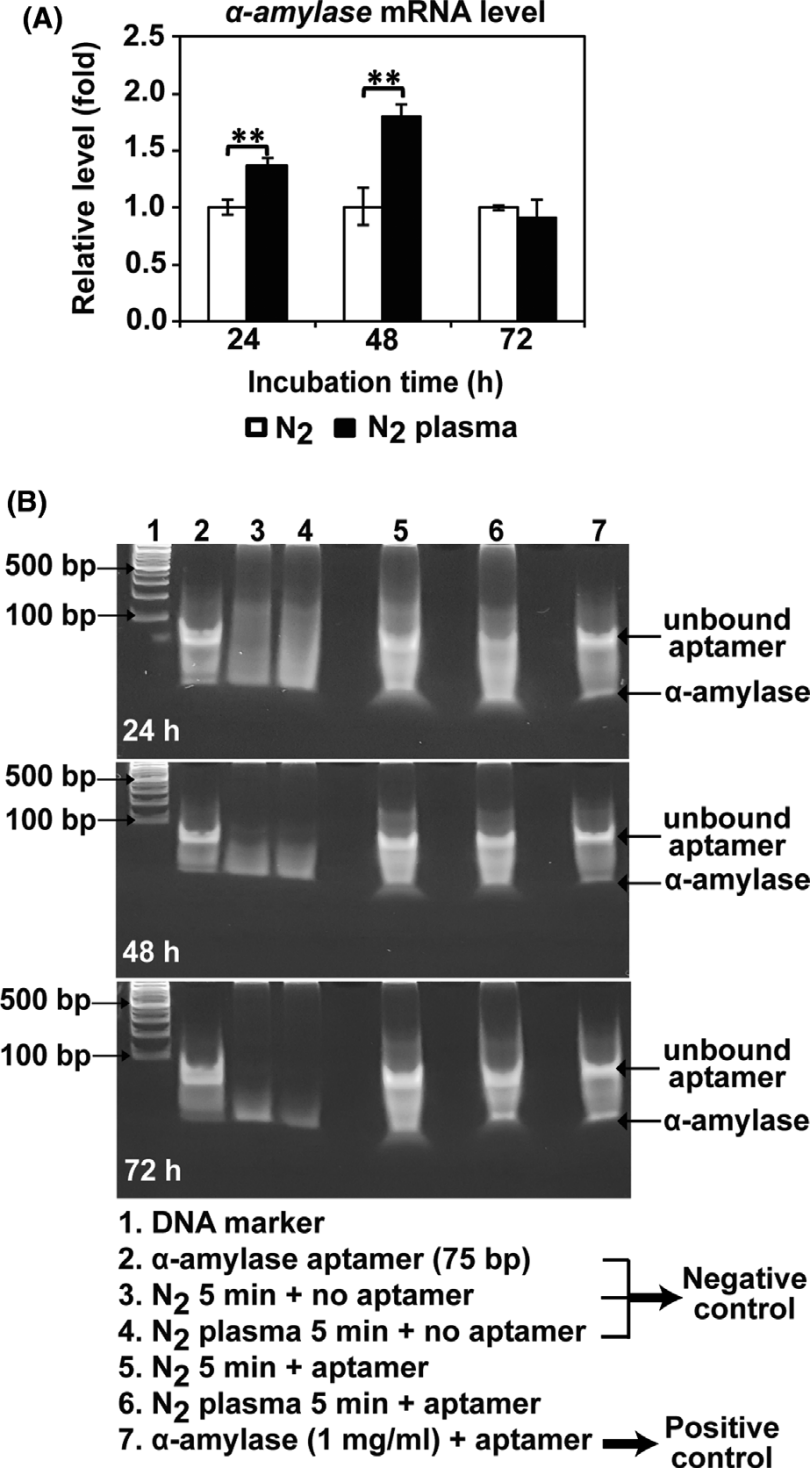
Intracellular expression of α‐amylase after plasma treatment. A. The mRNA level of α‐amylase in the fungal hyphae quantified by qPCR after 24, 48 and 72 h. Each value is the mean of nine replicate measurements; **P < 0.01. B. Intracellular α‐amylase detected by an α‐amylase aptamer in native polyacrylamide gel electrophoresis (8% in TBE; Tris/Borate/EDTA buffer, no sodium dodecyl sulphate).

The intracellular mRNA levels of α‐amylase were significantly higher in plasma‐treated samples (*P* < 0.01) than in the control (N_2_ gas only) after 24 and 48 h (Fig. 2A). An approximately 1.4‐ and 1.8‐fold increase was observed in the plasma‐treated samples after incubation for 24 and 48 h, respectively, while no significant difference was observed after 72 h (Fig. 2A). These results indicated that plasma treatment of fungal spores in PDB promoted transcription of the α‐amylase gene in hyphae that were grown for at least 24 h. No significant difference in the intensity of the α‐amylase protein band detected by the aptamer (Table 1) on native polyacrylamide gel electrophoresis was observed between control and plasma treatment after 24, 48 and 72 h (Fig. 2B). Although mRNA expression was increased after plasma treatment, protein translation from mRNA did not seem to be elevated. Therefore, whether plasma can significantly enhance the intracellular expression of α‐amylase was not resolved.

**Table 1:** Sequence of aptamer used to detect α‐amylase.

Primer name	Primer sequences
α‐amylase aptamer^a^	GGATACCTTAACGCCGCCTATTG **t**GAACGACG**t**GAA**t**AG**t**G**ttt**G**t**GGG**t**CCGGAG **tt** GCACCCG **t** C **t** CGAAA **t** C

^a^ Sequence is presented in the 5 to 3 direction. The underlined regions were derived from the primer or primer‐binding regions. Bold letters (**t**) indicate (E)‐5‐(2‐(N‐(2‐(N^6^‐adeninyl) ethyl)) carbamyl)‐uracil (U^ad^) (Minagawa *et al*., 2017).

**Fig. 3:**
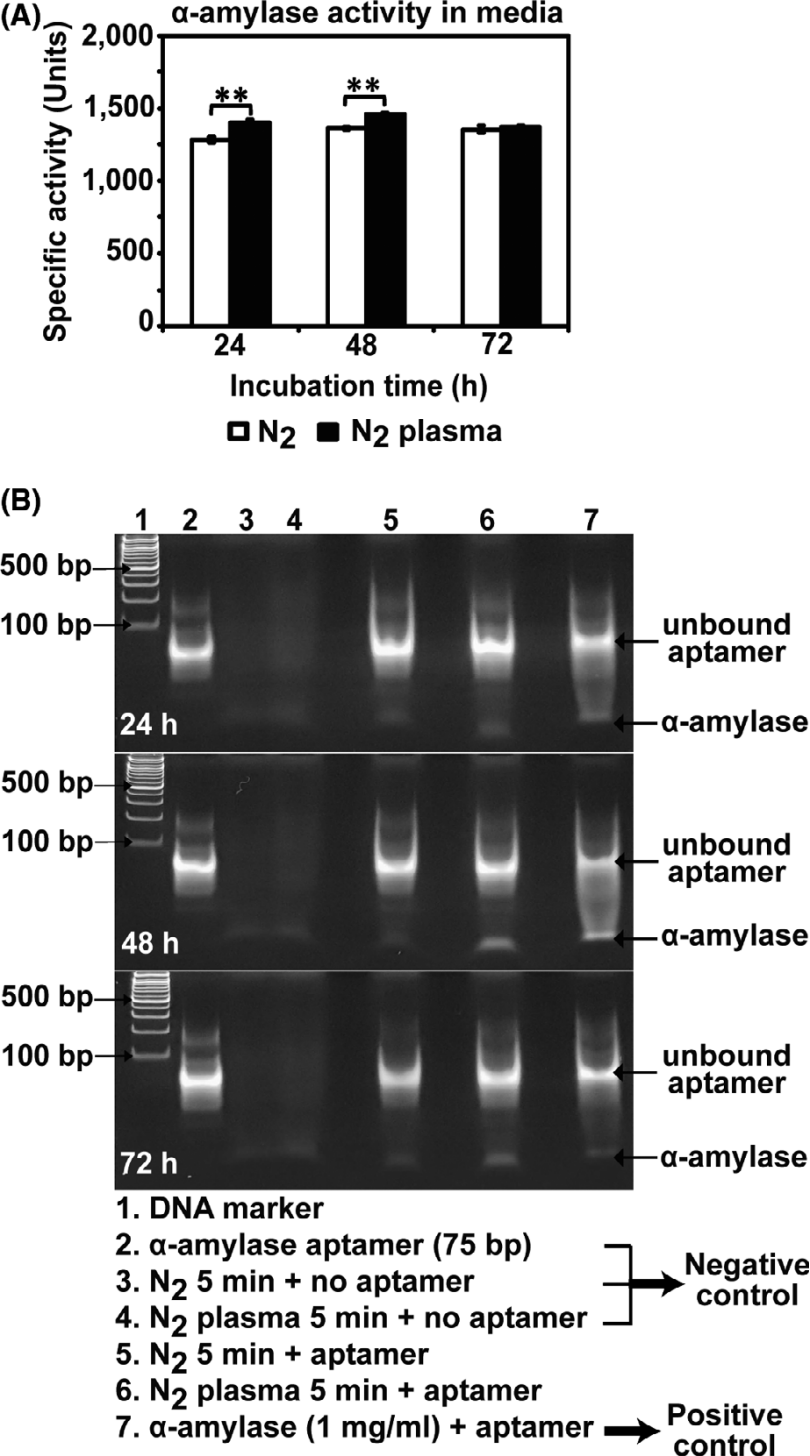
Analysis of extracellular secretion of α‐amylase after plasma treatment. A. Specific α‐amylase activity (units) measured in PDB media after 24, 48 and 72 h. Each value is the mean of three to nine measurements; **P < 0.01. B. The α‐amylase protein in PDB media detected by an α‐amylase aptamer in native polyacrylamide gel electrophoresis (8% in TBE, no sodium dodecyl sulphate).

The secretion of α‐amylase into the media was analysed by detecting the α‐amylase protein and quantifying its enzymatic activity in PDB. The specific activity of α‐amylase (enzyme activity expressed per milligram of total protein) in PDB media was increased by approximately 9.3% and 7.4% after 24 and 48 h of incubation, respectively, after plasma treatment of the spores (Fig. 3A). The band intensity of α‐amylase protein detected by the aptamer increased in the plasma‐treated samples after 48 and 72 h (Fig. 3B). This indicated that the plasma treatment of spores in PDB resulted in the enhanced secretion of α‐amylase from the fungal hypha after 24–48 h.

Plasma treatment of growing fungal hyphae (16 h), not spores, in PDB did not seem to cause any significant change in α‐amylase expression and extracellular secretion (Fig. S2). The level of intracellular α‐amylase mRNA and the level and activity of extracellular protein was not significantly different between the control (N2 gas only) and plasma‐treated samples at all times (Fig. S2).

### Long‐lived NO_X_ species may be critical for enhanced α‐amylase secretion

Since fungal spores submerged in PDB were treated with plasma, RONS generated in the PDB after plasma treatment could be a major factor affecting fungi (Thirumdas *et al*., 2018). We analysed the RONS in PDB solution during incubation following plasma treatment. Due to the limited available measurement tools, the analysis focused on hydrogen peroxide (H_2_O_2_) and nitric oxide species (NO_X_). We used the term NO_x_ because plasma‐generated NO can be rapidly oxidized to nitrite (NO_2_^−^) and nitrate (NO_3_^−^), which were also measured in our assay.

**Fig. 4:**
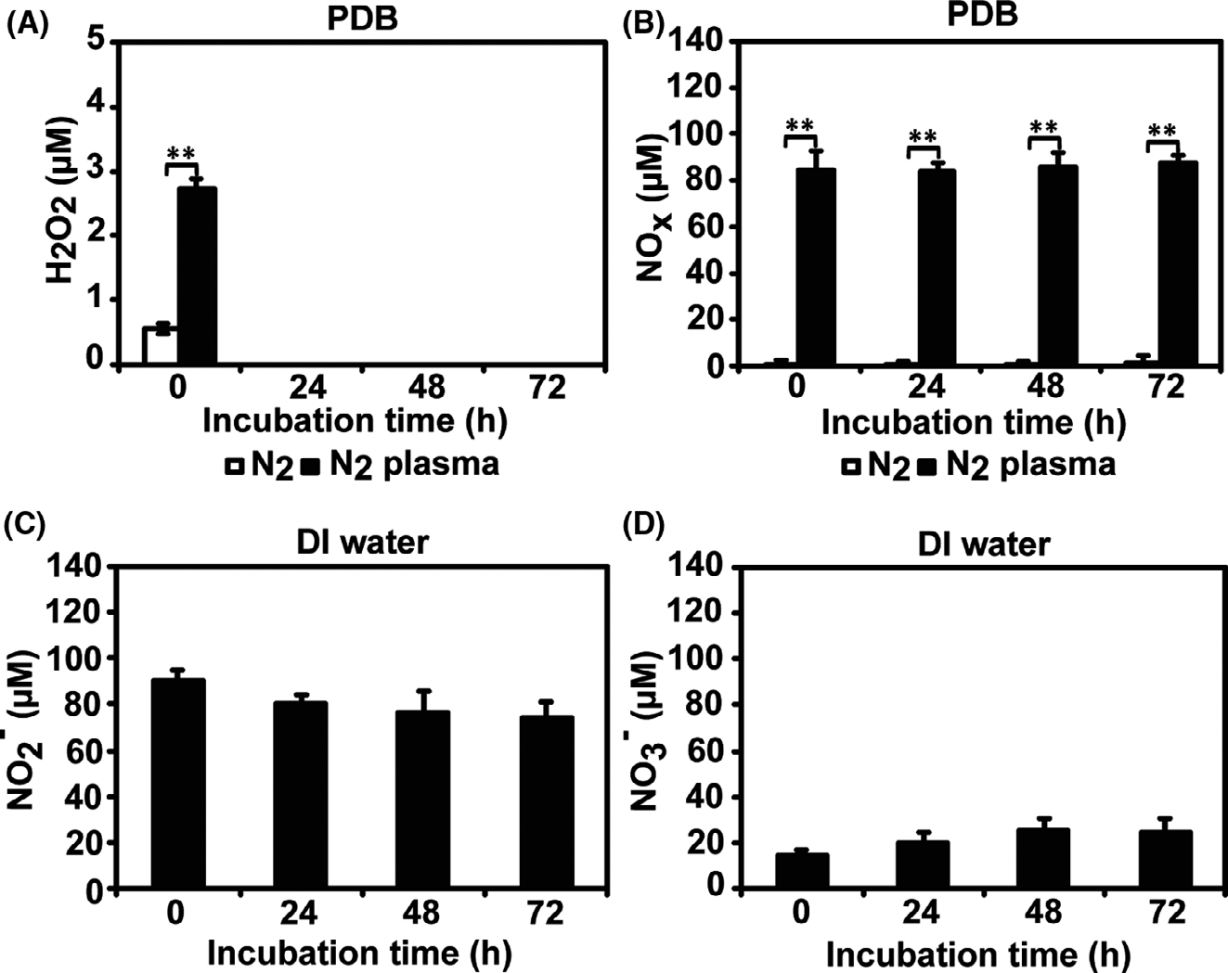
Reactive species analysis after plasma treatment. The concentrations of H_2_O_2_ (A) and NO_x_ (B) in PDB media were measured using assay kits after PDB was treated with N_2_ gas or plasma for 5 min and then incubated for 24, 48 and 72 h. The levels of NO_2_^−^ (C) and NO_3_^−^ (D) were estimated in DI water treated with plasma using a spectrophotometer after incubation for 24, 48 and 72 h. Each value represents the mean of 6–9 replicate measurements; **P < 0.01.

The levels of H_2_O_2_ and NO_x_ increased in the PDB solution following plasma treatment (Fig. 4A and B). The overall concentration of NO_X_ was markedly higher than that of H_2_O_2_ in PDB (2.73 vs 87 μM) during incubation for up to 72 h (Fig. 4A and B). H_2_O_2_ rapidly disappeared from PDB and was absent after 24 h, whereas the NO_X_ concentration was stably maintained in PDB during incubation for up to 72 h (Fig. 4A and B). Plasma‐generated NO (short‐lived species) can be oxidized to NO_2_^−^ and NO_3_^−^ (long‐lived species). All these species were detected by our assay, indicating that the total NO_X_ levels may not vary dramatically during incubation.

Analysis of the concentrations of NO_2_^−^ and NO_3_^−^ in culture media with a complex chemical composition, such as PDB, is complicated. Therefore, we analysed the NO_2_^−^ and NO_3_^−^ levels in plasma‐treated deionized (DI) water using circular dichroism spectrometry to examine PDB. During incubation for up to 72 h, the concentrations of NO_2_^−^ and NO_3_^−^ in plasma‐treated DI water were estimated as 74–90 μM and 15–25 μM, respectively (Fig. 4C and D). The levels of NO_2_^−^ decreased slightly during incubation, while the NO_3_^−^ level increased slightly (Fig. 4C and D). NO_2_^−^ and NO_3_^−^ were already detectable at 0 h, indicating that plasma‐generated NO was rapidly oxidized to NO_2_^−^ and then steadily oxidized to NO_3_^−^ (Fig. 4C and D). Oxidation of NO to NO_2_^−^ was likely faster than that from NO_2_^−^ to NO_3_^−^ (Fig. 4C and D). Our results suggested that long‐lived species, such as NO_2_^−^ and NO_3_^−^, could be the main reactive species present in PDB solution after plasma treatment. These species might influence α‐amylase secretion in *A. oryzae*.

**Fig. 5:**
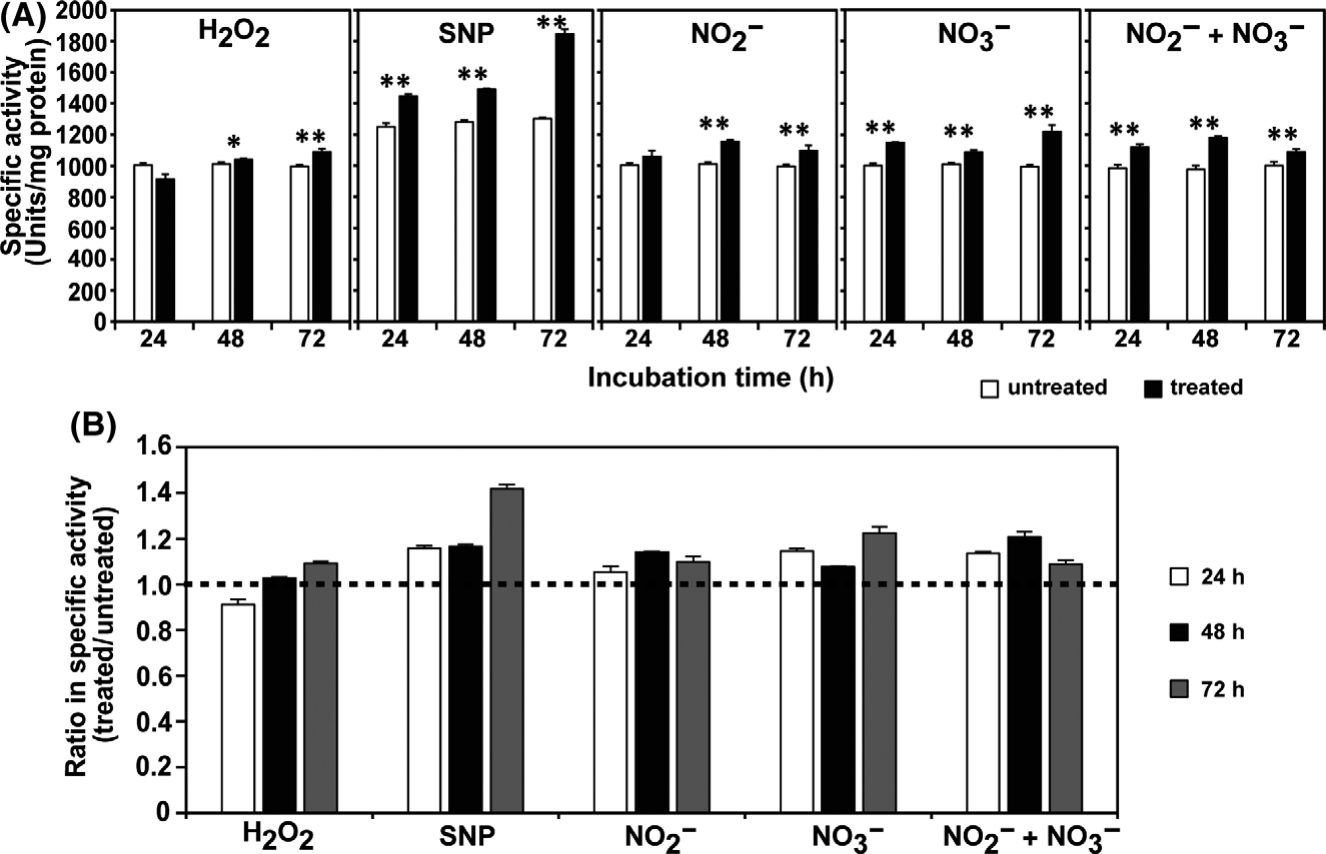
Effect of reactive species on the secretion of α‐amylase. A. The specific α‐amylase activity (units) measured in PDB media 24, 48 and 72 h after spores were exposed to individual species. B. The relative specific α‐amylase activity (units) of treated samples compared to that of untreated samples. The ratio that was calculated represented specific α‐amylase activity (units) detected in PDB treated with individual reactive species/ specific α‐amylase activity (units) detected in untreated PDB. Each value is the mean of three to nine replicate measurements; *P < 0.05, **P < 0.01.

Since plasma treatment promoted the secretion of α‐amylase in PDB, we analysed the effect of short‐ and long‐lived species on α‐amylase secretion in PDB. Secretion of α‐amylase was assessed by measuring the enzymatic activity in PDB after fungal spores were incubated in the presence of chemicals that produced H_2_O_2_, NO, NO_2_^−^ and NO_3_^−^ at the same concentrations as those found in plasma‐treated PDB after 5 min: 2.73 μM H_2_O_2_, 40 mM SNP (sodium nitroprusside) producing 84.8 μM NO, 90.49 μM NaNO_2_ and 14.87 μM NaNO_3_. The activity of α‐amylase in PDB was dramatically higher during 24–72 h of incubation with SNP, NaNO_2_ and NaNO_3_ than H_2_O_2_ (Fig. 5A and B). SNP induced greater enzyme secretion into the media than NO_2_^−^ and/or NO_3_^−^ at all incubation times (Fig. 5B). The findings indicated that NO can trigger enzyme secretion from fungal hyphae more efficiently than NO_2_^−^ and NO_3_^−^. This would occur because fresh NO is continuously generated from SNP during incubation. The α‐amylase activity was significantly higher after treatment with NO_2_^−^ and/or NO_3_^−^ (Fig. 5A). In addition, treatment with NO_3_^−^ alone or along with NO_2_^−^ resulted in a slightly greater increase in α‐amylase activity than treatment with NO_2_^−^ alone (Fig. 5A and B). These findings indicated that NO_3_^−^ may be more important for enzyme secretion than NO_2_^−^. This idea was also supported by the observation that treatment with a mixture of H_2_O_2_ and NO_2_^−^ (i.e. omission of NO_3_^−^) had less of an effect on enzyme secretion (Fig. S3a).

When the fungal spores were incubated in PDB containing 0.56 μM (control) or 2.73 μM H_2_O_2_, the intracellular α‐amylase mRNA levels were not significantly different after incubation for 24 h (Fig. S3b). After 48 and 72 h of incubation, the mRNA levels were significantly lower in the H_2_O_2_ treatment, indicating that H_2_O_2_ did not enhance transcription of α‐amylase. The α‐amylase mRNA levels were significantly higher after spores were exposed to 40 mM SNP compared to control (no SNP) at 24, 48 and 72 h of incubation (Fig. S3b).

### Accumulation of vesicles near hyphal tips after plasma treatment

**Fig. 6:**
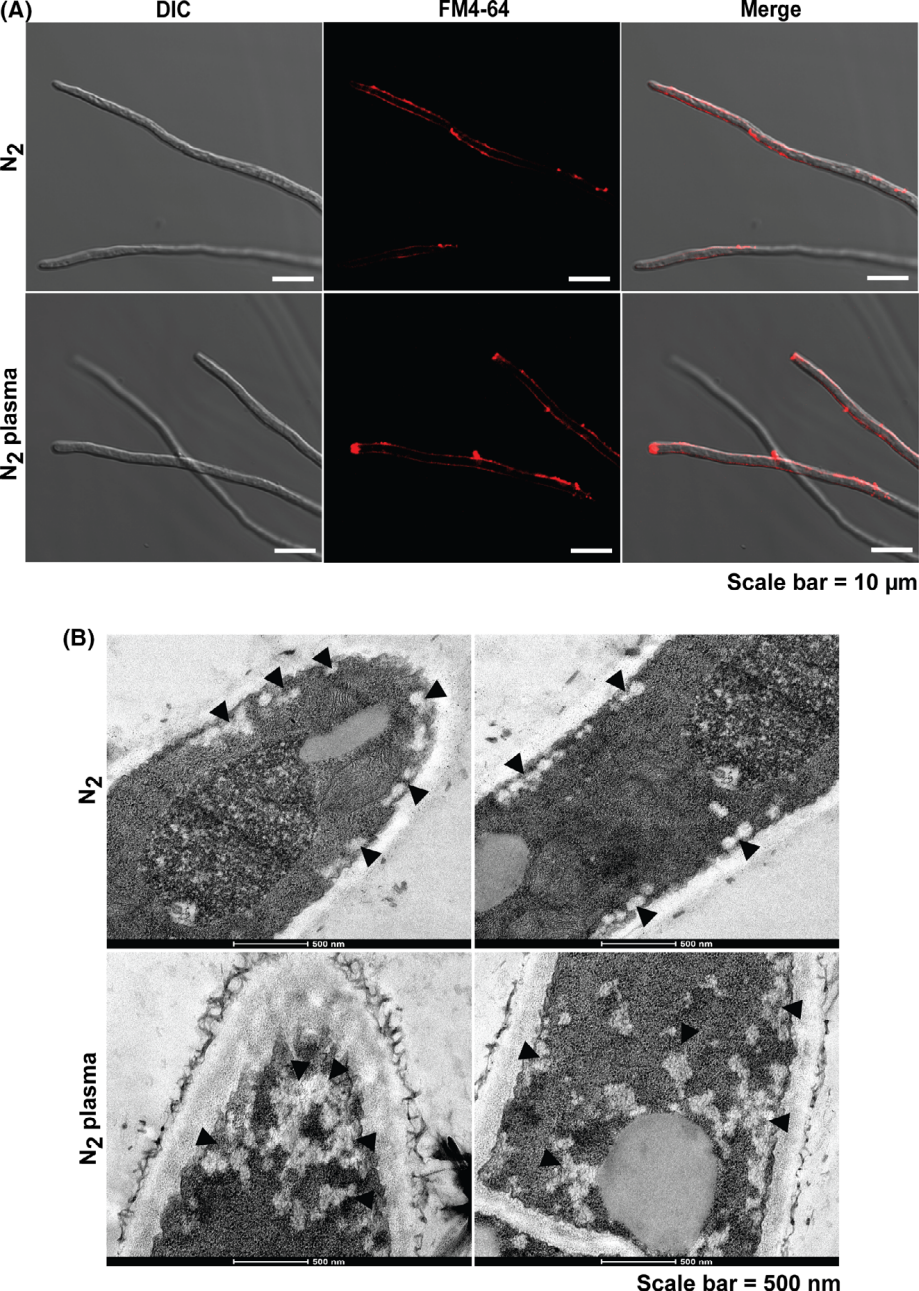
Visualization of secretory vesicles in the fungal hyphae after plasma treatment. A. The vesicles in the fungal hyphae stained with FM4‐64 after 24 h. DIC denotes differential interference contrast microscopy. B. The internal ultrastructure of the fungal hyphae after 24 h. The left and right panels show the tip and middle section of the fungal hyphae, respectively. The arrows indicate secretory vesicles.

Protein secretion in filamentous fungi occurs at the apical or sub‐apical hyphal regions through the transport of vesicles containing secretory proteins to the cell membrane (Hayakawa *et al*., 2011; Shoji *et al*., 2014). To determine how plasma‐derived, long‐lived species promote α‐amylase secretion, we first examined the localization of secretory vesicles containing enzymes in the fungal hyphal tips or sub‐apical regions. After 24 h, a higher level of fluorescence from vesicles stained with FM4‐64 was observed at the apical and sub‐apical hyphal tips in the plasma‐treated samples (Fig. 6A and Fig. S4). This finding indicated that long‐lived species, such as NO_2_^−^ and NO_3_^−^, generated in plasma may be associated with the promoted accumulation of protein (α‐amylase)‐containing vesicles at the hyphal tips and the sub‐apical area after 24 h. Fluorescence was mostly concentrated close to the cell surface and not in the cytosol, indicating that vesicles accumulated close to the plasma membrane (Fig. 6A and Fig. S4).

The vesicles in the fungal hyphae were also visualized by transmission electron microscopy. More vesicles were observed in the apical and sub‐apical regions of plasma‐treated samples than control samples after 24 h (Fig. 6B and Fig. S5).

### N_2_ plasma induces membrane depolarization and elevates intracellular Ca^2+^ levels

**Fig. 7:**
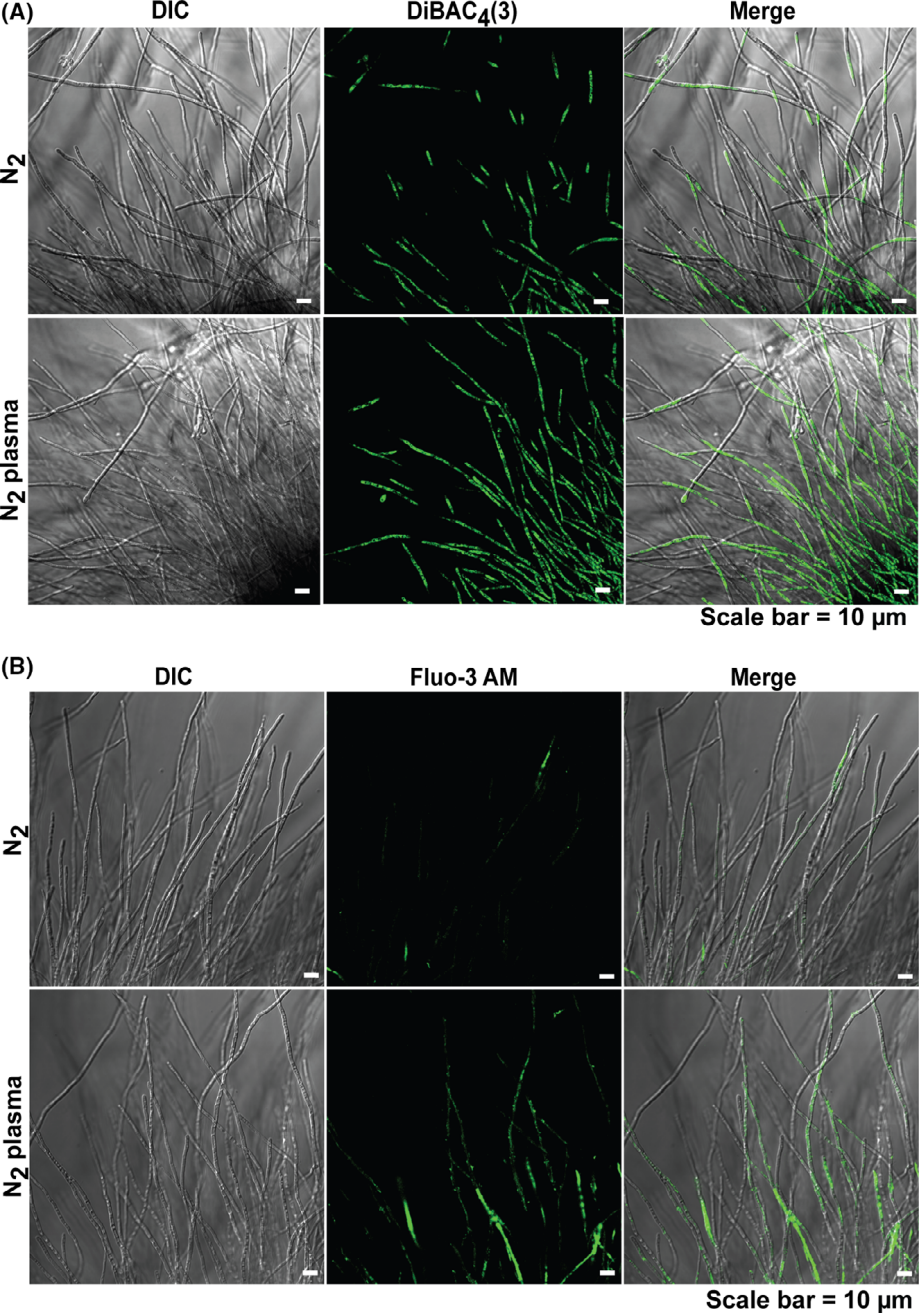
Analysis of membrane potential and intracellular Ca^2+^ levels in the fungal hyphae. A. Fungal hyphae labelled with DiBAC4(3). Fluorescence occurred upon membrane depolarization. B. Intracellular Ca^2+^ in fungal hyphae after 24 h following staining with Fluo‐3‐AM.

In nerve cells, membrane depolarization and subsequent increase in intracellular Ca^2+^ levels lead to the accumulation of secretory vesicles near the apical region (Liang *et al*., 2017). Accordingly, we analysed the membrane potential and intracellular calcium ion (Ca^2+^) in the fungal hyphae 24 h after plasma treatment. The fluorescence intensity indicating membrane depolarization was generally higher in fungal hyphae cultured for 24 h after N_2_ plasma treatment of spores than in control samples treated with N_2_ gas (Fig. 7A and Fig. S6). A larger area of the hyphae fluoresced in the plasma‐treated samples than in the control samples, indicating increased membrane depolarization in the fungal hyphae following plasma treatment (Fig. 7A and Fig. S6).

Staining of intracellular Ca^2+^ with Fluo‐3 AM was more pronounced in fungal hyphae 24 h after N_2_ plasma treatment of spores than in controls treated with N_2_ gas (Fig. 7B and Fig. S6). Fluo‐3 AM fluorescence was mostly observed in plasma‐treated samples, whereas fluorescence intensity in the controls was very low (Fig. 7B and Fig. S6). The plasma‐induced, long‐lived species present in the PDB might be involved in the depolarization of the hyphal membrane and subsequent elevation of intracellular Ca^2+^ levels.

### N_2_ plasma increases mRNA levels of genes involved in vesicle trafficking

**Fig. 8:**
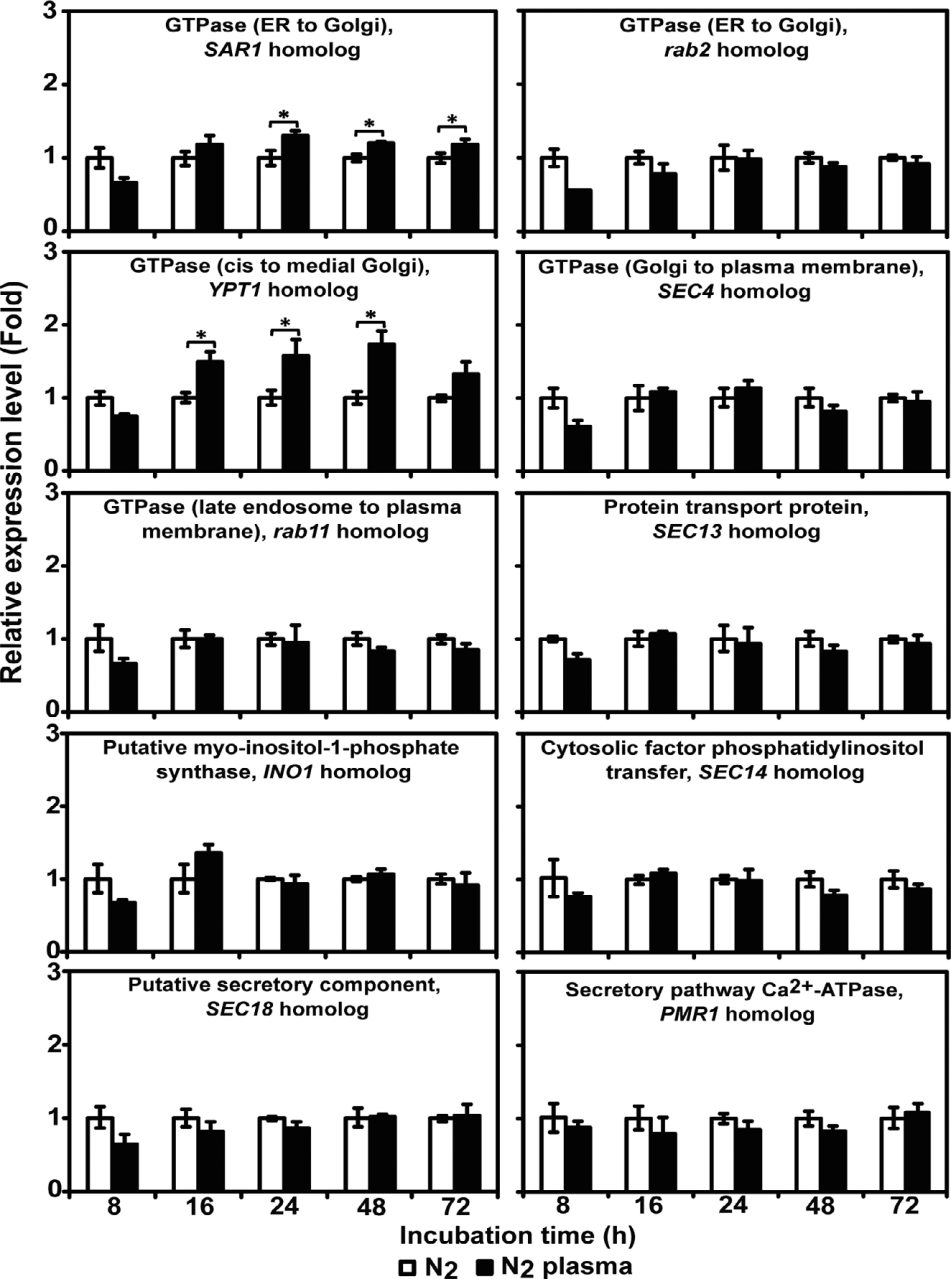
Transcription of genes involved in the fungal secretory pathway after plasma treatment. The mRNA levels of 10 secretion‐related genes in fungal hyphae were quantified by qPCR after 8, 16, 24, 48 and 72 h. Each value is the mean of three replicate measurements. *P < 0.05.

To elaborate the mechanism(s) for vesicle accumulation at hyphal and sub‐hyphal tips, the expressions of genes involved in the secretory protein pathway in *A. oryzae* (Sims *et al*., 2005) were examined. Among the 10 putative genes (Table 2), SAR1 homolog (GTPase controlling transport from the endoplasmic reticulum [ER] to the Golgi) and YPT1 homolog (GTPase controlling transport from cis to medial Golgi) showed significantly higher levels of transcription 24–48 h after plasma treatment of the spores (Fig. 8 and Table S1).

**Table 2:** List of primers used in qPCR.

Genes	Primer sequences
α‐Amylase	Forward‐ACTGGGTGGGATCATTGGTA
	Reverse‐ACAAGTGTAGGCCGGATCAC
Vesicle trafficking/transport	
GTPase (ER to Golgi), SAR1 homolog	Forward‐CGAAGTGAGCGGTATCGTTT
	Reverse‐CCCTTTCCTGTGGTCTGGTA
GTPase (ER to Golgi), rab2 homolog	Forward‐GGATCGCGTATTGTTCCAGT
	Reverse‐CCGTGTGATGGATTTGTACG
GTPase (cis to medial Golgi), YPT1 homolog	Forward‐TGATGGCAAGACAGTGAAGC
	Reverse‐TTGACACCCTCAGTGGCATA
GTPase (Golgi to plasma membrane), SEC4 homolog	Forward‐GAACTTGACGGAAAGCGTGT
	Reverse‐GCTCGACGTTTGAGAACCAT
GTPase (late endosome to plasma membrane), rab11 homolog	Forward‐GTCGGTGCCCTTCTTGTTTA
	Reverse‐TCGCATCAAGAGCAGATGTC
Protein transport protein, SEC13 homolog	Forward‐CAGTTTGGTGTATCGCATGG
	Reverse‐GCAAGAAGGCATCCACTCTC
Cellular export and secretion	
Putative myo‐inositol‐1‐phosphate synthase, INO1 homolog	Forward‐CTGTGACGGCCGGTATTATT
	Reverse‐GTCCTGGAAGGGAATGTTGA
Cytosolic factor phosphatidylinositol transfer, SEC14 homolog	Forward‐GTTGACTTTGCTCCGCTTTC
	Reverse‐CTTGCCCAGTTTCTCGATGT
Putative secretory component, SEC18 homolog	Forward‐CGAGATTGCAGGTCTTGTCA
	Reverse‐GGCTCGAAAGTTCCTCCTCT
Secretory pathway Ca^2+^‐ATPase, PMR1 homolog	Forward‐CCGTTCTTCGTGTCGGTAAT
	Reverse‐TCATCCTCCCCAAAAGTGTC
Reference gene	
18S ribosomal RNA	Forward‐GGAAACTCACCAGGTCCAGA
	Reverse‐AGCCGATAGTCCCCCTAAGA

## Discussion

Many reports have addressed the activation of cellular processes by NTAPP in humans and plants (Nastuta *et al*., 2011; Schmidt *et al*., 2015; Choi *et al*., 2017; Liu *et al*., 2017; Ito *et al*., 2018; Shi *et al*., 2018; Park *et al*., 2019). However, the activation of microbial cell functions by plasma has been demonstrated in a few cases such as fungal spore germination (Veerana *et al*., 2019), production of heterologous enzyme (Farasat *et al*., 2018), and secretion of fungal proteases (Gao *et al*., 2020). Our study provides additional evidence for the activation of microbial cellular processes by plasma (or plasma‐generated long‐lived species). The present and prior findings by other groups may be useful for improving the function of beneficial microorganisms. However, there could be dangerous consequences with respect to harmful pathogenic microorganisms. Thus, careful application and complete control of microbial species and treatment conditions are required. The majority of microorganisms are beneficial or neutral to humans. The efficient utilization of these resources may also be important.

Two mechanisms for enhanced enzyme secretion by plasma can be proposed. First, the enhanced enzyme secretion may be a consequence of the enhanced germination of fungal spores after plasma treatment. After spore germination was increased, further hyphal development accelerated and enzyme secretion from actively growing fungal hyphae increased. We did not systematically examine this hypothesis in this study. However, no significant difference between control and plasma treatment was observed in the dry weight of fungal mycelia after 3 days in our preliminary data (Fig. S8). This observation indicates that enhanced spore germination may not be closely associated with the promotion of hyphal growth. The second hypothesis is that plasma‐derived, long‐lived species in the media could play a critical role in regulating enzyme secretion because plasma was applied to fungal spores and enzyme secretion was checked after 24 h (at the hyphal stage). The observations indicated that long‐lived nitrogen species, such as NO_2_^−^ and NO_3_^−^, could affect α‐amylase secretion. In plasma‐treated PDB, NO was produced in a short burst and then disappeared over time. Therefore, the effect of NO on enzyme secretion may be minimal in plasma‐treated PDB.

One drawback of our study is that we did not have data on the levels of NO_2_^−^ and NO_3_^−^ in the PDB media after plasma treatment. Although direct measurement of NO_2_^−^ and NO_3_^−^ concentrations in PDB is not easy, our estimated levels of NO_2_^−^ and NO_3_^−^ in plasma‐treated water did not seem to be significantly different from NOx levels measured in plasma‐treated PDB. This suggests that our extrapolation of NO_2_^−^ and NO_3_^−^ levels in plasma‐treated PDB using that in plasma‐treated water are acceptable. Only NO_2_^−^ and NO_3_^−^ were included as long‐lived species in this study. However, other species could be generated in PDB media after plasma treatment or other unknown factors could play a role in protein secretion. Further studies are required.

**Fig. 9:**
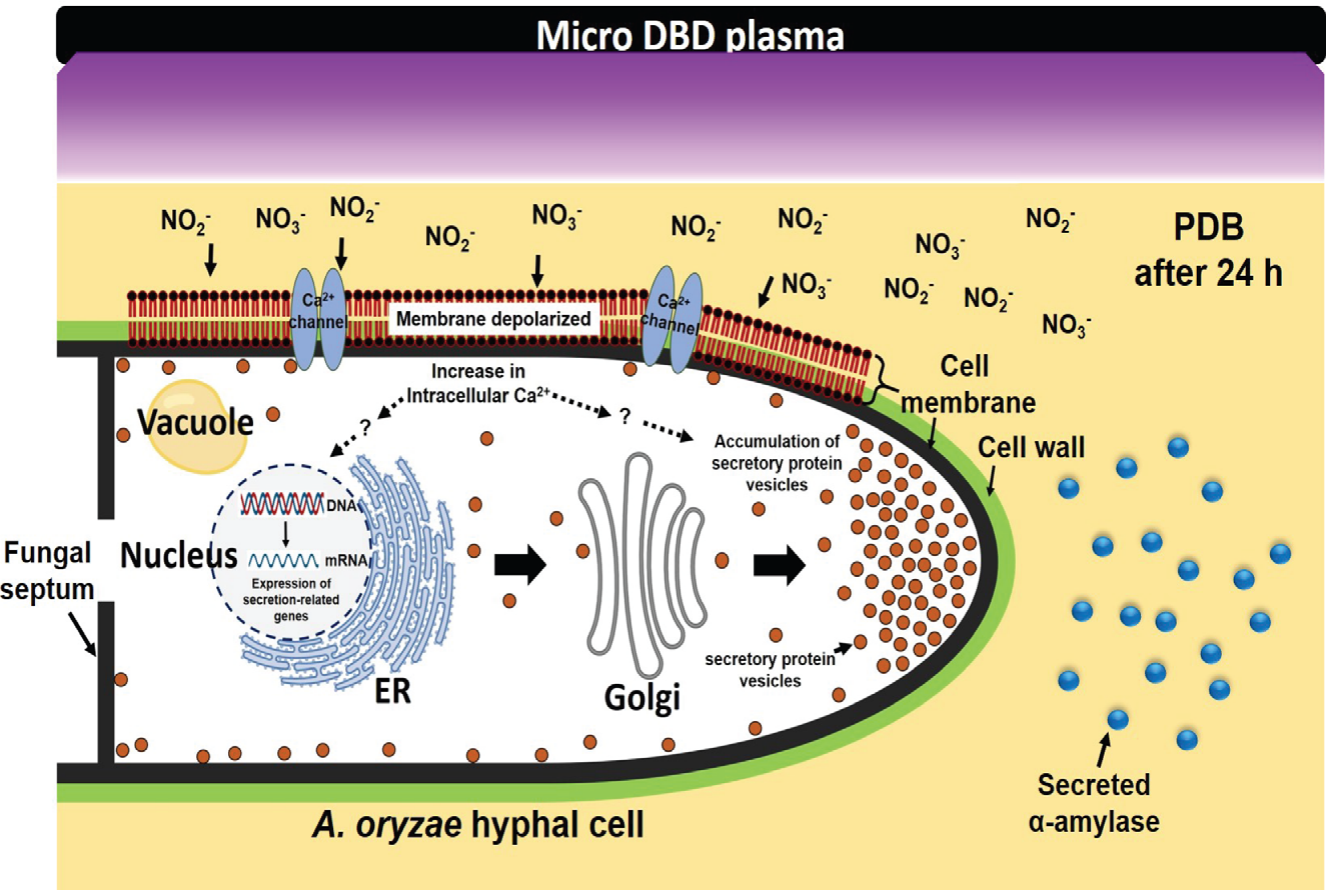
Proposed model for the mechanism(s) of plasma‐activated protein secretion in *A. oryzae*. Secretion of α‐amylase from fungal hyphae at least 24 h after plasma treatment on fungal spores was modelled.

Our results suggest that plasma‐generated, long‐lived species are closely associated with the increased accumulation of vesicles near the hyphal tips and sub‐apical area. Increased accumulation of secretory vesicles in plasma‐treated samples may be related to membrane depolarization and intracellular Ca^2+^ levels as demonstrated in neurons and endocrine cells (Bose *et al*., 2015; Liang *et al*., 2017). Plasma‐generated, long‐lived NO_2_^−^ and NO_3_^−^ can directly encounter and interact with the fungal cell surface. This may cause changes in the physical and chemical properties of the cell membrane. Subsequently, ion channel proteins in the membrane could be activated or inactivated, leading to membrane depolarization. Membrane depolarization and activation of Ca^2+^ channels by reactive oxygen (H_2_O_2_) and nitrogen species have often been observed in neurons, plants and microbes (Pei *et al*., 2000; Popa *et al*., 2010; Zhong *et al*., 2013). A recent study showed that the short‐lived reactive species (OH) generated via He‐plasma irradiation in 4‐(2‐hydroxyethyl)‐1‐piperazineethanesulfonic acid‐buffered saline solution triggered Ca^2+^ influx through channels and increased Ca^2+^ levels in fibroblast cells (Sasaki *et al*., 2016). These studies demonstrate the importance of short‐lived reactive species in membrane depolarization and Ca^2+^ influx. However, our study shows that plasma‐derived, long‐lived species of NO_2_^−^ and NO_3_^−^ can be more important in triggering membrane depolarization through the activation of the Ca^2+^ influx system (Fig. 9).

The increased expression of GTPases involved in early vesicle trafficking from the ER to the Golgi in the plasma‐treated samples may be resulted from elevation of intracellular Ca^2+^ levels. Increased intracellular Ca^2+^ concentration in yeasts and filamentous fungal cells regulates a wide range of cellular processes, such as cell cycle progression, sporulation, spore germination, oriented hyphal tip growth, hyphal branching, gene expression, secretion and circadian rhythms (Liu *et al*., 2015). In addition, several studies have indicated that Ca^2+^ is required for the constitutive secretory trafficking from the ER to the Golgi (Beckers and Balch, 1989), early endosome fusion (Colombo *et al*., 1997), yeast homotypic vacuole fusion (Peters and Mayer, 1998) and triggering of the final step of vesicle fusion (Katz and Miledi, 1968; Schneggenburger and Neher, 2000). Therefore, the presently observed increase in intracellular Ca^2+^ levels could trigger the expression of genes involved in secretory protein pathways.

Finally, chemical changes or reactive species (short and long‐lived) generated in PDB media after plasma treatment may not significantly affect the molecular structure of secreted α‐amylase. A slightly low pH (close to 5) was maintained in PDB media after plasma treatment during the incubation period (Fig. S9). However, fungal α‐amylase has a wide optimal pH range of acidic to neutral for activity (Saranraj and Stella, 2013). Therefore, a pH of approximately 5 should not influence enzymatic activity. In addition, a simulation performed on α‐amylase molecular structure constructed using amino acid sequences showed that the backbone fluctuation (RMSD), stability and folding (SASA), compactness (Rg) and flexibility (RMSF) of the α‐amylase protein were not dramatically modified in the presence of H_2_O_2_, NO_2_^−^ and NO_3_^−^ at the concentration measured in plasma‐treated PDB and water (Fig. S10). These observations suggest that α‐amylase may be structurally and functionally stable after secretion into the plasma‐treated media, making the application of plasma more reliable and safer.

## Conclusions

Improved production and secretion of useful enzymes by microorganisms is a common industrial target. Our study demonstrates that NTAPP can be a potential tool for enhancing protein secretion from filamentous fungi. Plasma‐derived, long‐lived species produced in the media can induce membrane depolarization and increase Ca^2+^ influx into the fungal cells, leading to the activation of secretory protein vesicle trafficking and accumulation of secretory vesicles in the apical and sub‐apical hyphal regions. The data demonstrate the activation of the protein secretory pathway by plasma in filamentous fungi.

## Experimental procedures

### Fungi and growth conditions

The fermenting fungus *A. oryzae* (strain KACC47488), also known as the koji mould, was graciously provided by the Korean Agriculture Type Collection at the National Agrobiodiversity Center (Wanju‐gun, Jeollabuk‐do, Republic of Korea). The fungi were propagated and maintained on potato dextrose agar (PDA) medium or PDB (MB cell, Los Angeles, CA, USA) at 30°C in the dark.

### Treatment of fungal spores with nitrogen plasma

Fungal spores were treated with NTAPP using a micro DBD plasma unit equipped with a burst pulse‐type high‐voltage inverter (Fig. 1). The structure and organization of the electrodes and the cooling system have been previously described (Veerana *et al*., 2019). To generate plasma, nitrogen (N_2_) gas was injected into the device at a flow rate of 1 l min^−1^. An input voltage of 1.2 kV and current of 50–63 mA were used.

*Aspergillus oryzae* spores were harvested from 1‐week‐old culture plates. Sterile phosphate‐buffered saline (PBS; 15 ml) was added to the plates and the fungal mycelia were scraped using a spreader. The fungal suspension was filtered through four layers of sterile miracloth (Calbiochem, Darmstadt, Germany). After centrifugation of the filtered suspension at 3134 × *g* for 5 min, the supernatant was discarded. The spore pellet was resuspended in PDB solution, adjusting the concentration to 5 × 10^6^ spores per ml. Fifteen millilitres of the suspension were placed in a 90 mm‐diameter petri dish and exposed to plasma from a distance of 10 mm for 5 min (Fig. 1). Spores exposed to N_2_ gas were used as controls. Following treatment, the spore suspension was transferred to a 100 mL Erlenmeyer flask and incubated at 30°C with shaking for the indicated times (Fig. 1).

### Analysis of α‐amylase using aptamers

To analyse the expression and secretion of α‐amylase in *A. oryzae*, the protein levels inside the cells (expression) and the levels secreted into the media were qualitatively estimated using an α‐amylase specific aptamer (Table 1).

Spores (5 × 10^6^ fungal spores per ml, 15 ml) suspended in PDB were treated with N_2_ gas (control) and plasma for 5 min. The treated spores were incubated at 30°C with shaking for 24, 48 and 72 h. After incubation, the fungal culture was passed through a layer of sterile miracloth. For analysis of the secreted α‐amylase, the filtrate was collected in a new conical tube and stored at 4°C. The fungal mycelia left on the miracloth after filtration were thoroughly washed with sterile water to remove bound extracellular proteins and other contaminants. After completely removing the water from the fungal mycelia using a paper towel, the total protein was extracted as described previously (Nandakumar and Marten, 2002). The mycelia were ground in liquid nitrogen and then 1 ml lysis buffer (20 mM Tris‐HCl, pH 7.6, 10 mM NaCl and 0.5 mM deoxycholate) was added with glass beads. The mixture was vortexed four times and centrifuged at 6000 × *g* at 4°C for 10 min. The supernatant was collected and stored at −20°C. The total protein concentration was measured using a protein assay kit (Bio‐Rad, Hercules, CA, USA).

The intracellular and secreted α‐amylase were detected using an aptamer specific to *A. oryzae* α‐amylase (Table 1) (Minagawa *et al*., 2017). Extracts of total protein and media filtrate (5 μl each) were incubated with or without 3 μl of 10 μM α‐amylase aptamer at room temperature for 3 h in the dark. A commercial solution of *A. oryzae* α‐amylase (1 mg ml^−1^; Sigma‐Aldrich, St. Louis, MO, USA) was used as a positive control. Native (no sodium dodecyl sulphate) polyacrylamide gel electrophoresis was performed (8% in 1 × TBE; Tris/Borate/EDTA buffer) using a Mini‐PROTEAN^®^ Tetra Vertical Electrophoresis Cell (Bio‐Rad). α‐Amylase was detected using a ChemiDoc™ MP Imaging System (Bio‐Rad).

### Assay for α‐amylase activity

To measure α‐amylase activity in the culture media, 100 μl of serially diluted media was added to 100 μl of 1% (w/v) soluble starch in 0.1 M acetate buffer (pH 5.6). A separate blank (media) was prepared for each sample to eliminate the non‐enzymatic release of sugars. The reaction mixture was incubated at 50°C for 30 min (Sahnoun *et al*., 2012). The liberated reducing sugars (maltose equivalents) that were the products of the amylase reaction were detected using the 3, 5‐dinitrosalicylic acid method (Bernfeld, 1955) and quantified using a maltose standard curve. One unit (U) of amylase activity was defined as the amount of enzyme that released 1 μg of maltose (as reducing sugar equivalents) per minute under the assay conditions. Specific activity was expressed as amylase activity (U) per mg of protein.

### Measurement of H_2_O_2_, NO_x_, NO_2_^−^ and NO_3_^−^ levels in PDB and water

To analyse H_2_O_2_ and NO_x_ levels in background media, 15 ml PDB was treated with N_2_ gas (control) or plasma for 5 min each. Following treatment, concentrations of H_2_O_2_ and NO_x_ were measured at indicated times during incubation using an Amplex™ Red Hydrogen Peroxide/Peroxidase Assay Kit (Molecular Probes, Eugene, OR, USA) and a QuantiChrom™ Nitric Oxide Assay Kit (BioAssay Systems, Hayward, CA, USA), respectively, following the manufacturer’s protocols.

Levels of NO_2_^−^ and NO_3_^−^ were estimated in plasma‐treated deionized (DI) water. The estimates were not made in PDB solution due to technical difficulties. The levels of NO_2_^−^ and NO_3_^−^ in plasma‐treated DI water could provide a rough estimate of the same in plasma‐treated PDB. Following plasma treatment, the amount of NO_2_^−^ and NO_3_^−^ in plasma‐treated DI water was analysed by measuring the absorbance at 230–240 nm and 200–210 nm, respectively, using a model j‐815 circular dichroism spectrometer (JASCO, Easton, MD, USA) (Oh *et al*., 2018).

### Treatment of fungal spores with exogenous H_2_O_2_, NO_x_, NO_2_^‐^ and NO_3_^‐^

To understand the effect of short‐lived and long‐lived reactive species on the secretion of α‐amylase in *A. oryzae*, the fungal spores were submerged in PDB solution (5 × 10^6^ spores ml^−1^, 15 ml) containing H_2_O_2_ (final concentration 2.73 μM), SNP (final concentration 40 mM producing approximately 85 μM NO_x_), NaNO_2_ (final concentration 90.49 μM) or NaNO_3_ (final concentration 14.87 μM). These treatments generated a similar level of the individual species measured in PDB or DI water immediately after plasma treatment. The spore suspension was incubated at 30°C with shaking for the indicated times. At each time point, fungal culture was passed through a layer of miracloth and the filtrate media was collected to analyse α‐amylase activity. The assay for α‐amylase activity was performed as described above.

### Membrane potential assay

To analyse fungal membrane potential, spores of *A. oryzae* suspended in PDB (5 × 10^6^ fungal spores per ml, 15 ml) were treated with N_2_ gas (control) or plasma for 5 min. After treatment, the spore suspension was incubated at 30°C for 24 h with shaking. The fungal hyphae were washed with sterile PBS and incubated in 1 ml of PBS containing 50 μg of bis‐(1,3‐dibutylbarbituric acid) trimethine oxonol (DiBAC4(3); Invitrogen, Carlsbad, CA, USA) for 1 h at 4°C in the dark. Subsequently, the fungal hyphae were washed with PBS and the fluorescence was analysed at 490 nm (excitation) and 516 nm (emission) using a confocal laser scanning microscope (FV‐1000 MPE spectra; Olympus Corporation, Tokyo, Japan).

### Measurement of intracellular calcium

Fluo‐3 AM fluorescent calcium indicator (Invitrogen) was used to detect intracellular calcium in the hyphae of *A. oryzae*. Fungal spores (5 × 10^6^ per ml, 15 ml) in PDB were treated with N_2_ gas (control) or plasma for 5 min and incubated at 30°C with shaking for 24 h. The fungal hyphae were washed with PBS and incubated in 10 mM Fluo‐3 in PBS at 30°C for 30 min. After incubation, the fungal hyphae were washed with PBS and the fluorescence was detected at 506 nm (excitation) and 526 nm (emission) nm using the confocal laser scanning microscope.

### Vesicle staining

Spores of *A. oryzae* (5 × 10^6^ per ml, 15 ml) suspended in PDB were treated with N_2_ gas (control) or plasma for 5 min. After treatment, the spore suspension was incubated at 30°C with shaking for 24 h. Fungal hyphae were collected and washed with PBS. After washing, the vital dye FM4‐64 (Invitrogen) prepared in PBS (25 μM) was added to the samples and incubated at room temperature for 30 min in the dark. Fluorescence intensity was analysed at 514 nm (excitation) and 670 nm (emission) nm using the confocal laser scanning microscope.

### Visualization of vesicles in fungal hyphae by transmission electron microscopy

Fungal spores in PDB (5 × 10^6^ fungal spores per ml, 15 ml) were treated with N_2_ gas (control) or plasma for 5 min and cultured at 30°C with shaking for 24 h. The fungal hyphae were collected, washed twice with PBS and fixed in Karnovsky’s fixative [2% (v/v) paraformaldehyde and 2% (v/v) glutaraldehyde in 1 × PBS] at 4°C overnight. The fungal hyphae were washed three times with 0.05 M sodium cacodylate buffer and fixed with 1% (v/v) osmium tetroxide at 4°C for 2 h. The hyphae were washed three times with distilled water and dehydrated by serial incubations in 30, 50, 70, 80, 90 and 100% ethanol (three times). One millilitre of 100% propylene oxide was added to the dehydrated fungal hyphae and incubated at room temperature for 15 min. This step was repeated once. After the propylene oxide was removed, a 1:1 (v/v) mixture of propylene oxide and Spurr’s resin (cycloaliphatic epoxide resin ERL‐4221: D.E.R. 736 Epoxy resin: nonenyl succinic anhydride modified: dimethylaminoethanol in the ratio of 2:1.2:5.2:0.06) was added to the tube and incubated at room temperature for 2 h. After centrifugation at 9391 × *g* for 5 min, the resin mixture was discarded and a new mixture (1:2, v/v) of propylene oxide and Spurr’s resin was added and incubated at room temperature for 3 h with gentle mixing followed by removal of the resin mixture after centrifugation at 9391 × *g* for 5 min. Fresh Spurr’s resin (1 ml) was added and mixed with the fungal hyphae by rocking at 4°C overnight. The next day, the resin was removed by centrifugation and fresh Spurr’s resin was added to the tube, which was then rocked at room temperature for 3 h. The tubes were incubated at 70°C overnight to induce polymerization. The polymerized resin blocks were sectioned using an ultramicrotome (Leica, Solms, Germany), and the sectioned specimens were examined by transmission electron microscopy (JEOL, Tokyo, Japan).

### Quantitative PCR analysis

To measure the mRNA levels of α‐amylase and genes associated with protein secretion, spores in PDB media (5 × 10^6^ per ml, 15 ml) were treated with N_2_ gas (control) or plasma for 5 min and cultured at 30°C with shaking. During incubation, fungal samples were harvested at specific time points, washed twice with DI water, and stored at –80°C to extract total RNA using an RNAiso Plus kit (TaKaRa Bio, Shiga, Japan) according to the manufacturer’s instructions. RNA concentration was measured using a NanoDrop spectrophotometer (Biotek Instruments, Winooski, VT, USA). Equal amounts of RNA (0.1 μg) were used for cDNA synthesis, which was performed using the ReverTra Ace qPCR RT Master Mix with gDNA Remover (Toyobo, Osaka, Japan) according to the manufacturer’s instructions. The α‐amylase and ten putative genes related to protein secretion in *A. oryzae* were amplified and quantified at every thermal cycle using the iQ SYBR Green Supermix (Bio‐Rad) and a CFX96TM real‐time RT‐PCR system (Bio‐Rad). Conditions for thermal cycling were as follows: 95°C for 3 min, 40 cycles of 95°C for 10 s and 60°C for 30 s. Relative mRNA levels were expressed as a ratio of the expression levels of a reference gene (18S ribosomal RNA). The cycle threshold (Ct) value was determined. The difference in Ct values between N_2_ gas (control) and plasma‐treated samples was used to calculate the relative target gene expression level as follows: Ratio = (2)^∆Ct target (control‐sample)^/(2)^∆Ct reference (control‐sample)^ (Livak and Schmittgen, 2001). Primer sequences of α‐amylase and ten putative genes related to the secretory pathway in *A. oryzae* (Sims *et al*., 2005) are listed in Table 2. An average of three replicate measurements were obtained for each experiment.

### Statistical analysis

All data were collected from at least three replicates. The results are presented as the average. Statistical analysis of the data was performed using Student's *t*‐test. A statistically relevant difference was denoted by *P* < 0.05 (*) or *P* < 0.01 (**).

## Conflict of interest

The authors declare no competing interests.

## Supporting information

Supporting Information
