# Supplementary material for: Plasma‐mediated enhancement of enzyme secretion in Aspergillus oryzae
Source: Microb Biotechnol. 2020 Nov 5;14(1):262–76. doi: 10.1111/1751-7915.13696 (PMC7888467; doi:10.1111/1751-7915.13696)
Supplement: Supplementary file 1 — Supporting Information [file MBT2-14-262-s001.docx]

Supplementary Information

**Plasma-mediated enhancement of enzyme secretion in *Aspergillus oryzae***

*Mayura Veerana^1^, Sarmistha Mitra^1^, Se-Hoon Ki^,^**^2^, Soo Min Kim^3^, Eun-Ha Choi^1,2^, Taek Lee^3^, Gyungsoon Park^1,2*^*

^1^Department of Plasma Bioscience and Display, Kwangwoon University, Seoul, 01897, Korea

^2^Department of Electrical and Biological Physics, Kwangwoon University, Seoul, 01897, Korea

^3^Department of Chemical Engineering, Kwangwoon University, Seoul, 01897, Korea

* Corresponding Author

Gyungsoon Park

Phone: +82-2-940-8324

Fax: +82-2-940-5664

Email: gyungp@kw.ac.kr

**Supplementary Methods**

**Treatment of *A. oryzae* hyphae grown for 16 h with micro DBD plasma**

Fungal spores in PDB media (5x10^6^ spores/ml,15 ml) were incubated at 30 ºC with shaking for 16 h. After 16 h, the germinated fungal hyphae were exposed to N_2_ gas (control) or micro DBD plasma using N_2_ as a feeding gas for 5 min. After exposure, the treated fungal hyphae were further incubated at 30°C with shaking for indication time.

**Simulation analysis for the molecular dynamics of α-amylase in the presence of reactive species.**

In order to understand the effect of reactive species on the α-amylase molecular structure, a simulation analysis was performed on 3-dimentional molecular structure of α-amylase constructed using amino acid sequence with or without the mixture of H_2_O_2_, NO_2_^-^ and NO_3_^-^. The amino acid sequence of α-amylase protein of *A. oryzae* was obtained from National Center for Biotechnology Information (accession number P0C1B4). The protein stability and conformational change were analyzed by simulation in the presence of H_2_O_2_, NO_2_^-^ and NO_3_^-^ at the concentrations measured in PDB and water 0 h and 24 h after plasma treatment; 2.73 µM H_2_O_2_, 90.49 µM NO_2_^-^ and 14.87 µM NO_3_^-^ at 0 h and 80.15 µM NO_2_^-^ and 20.17 µM NO_3_^-^ at 24 h. The molecular dynamics simulations of α-amylase were performed using YASARA Dynamic software (YASARA biosciences GmbH, Vienna, Austria) as previously described (Hosen *et al*., 2019).


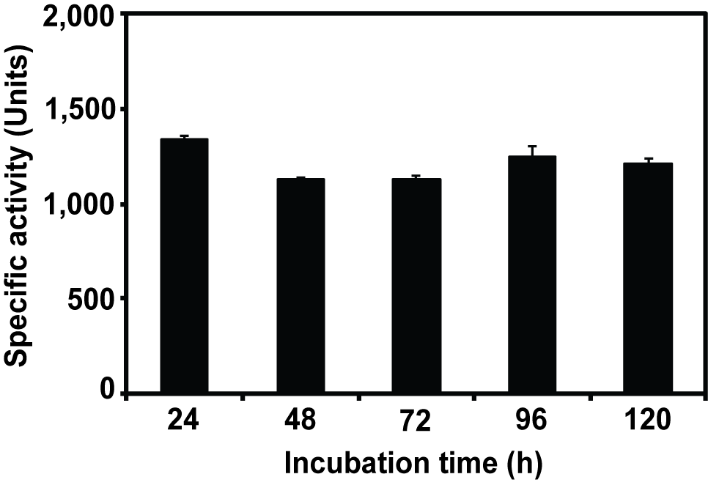


**Supplementary Figure S1**. **The α-amylase activity in PDB media.**

The level of extracellular α-amylase was assessed during culture of *A. oryzae* by measuring the specific α-amylase activity (units) in PDB media. Each value is the mean of 3-9 replicate measurements.

**
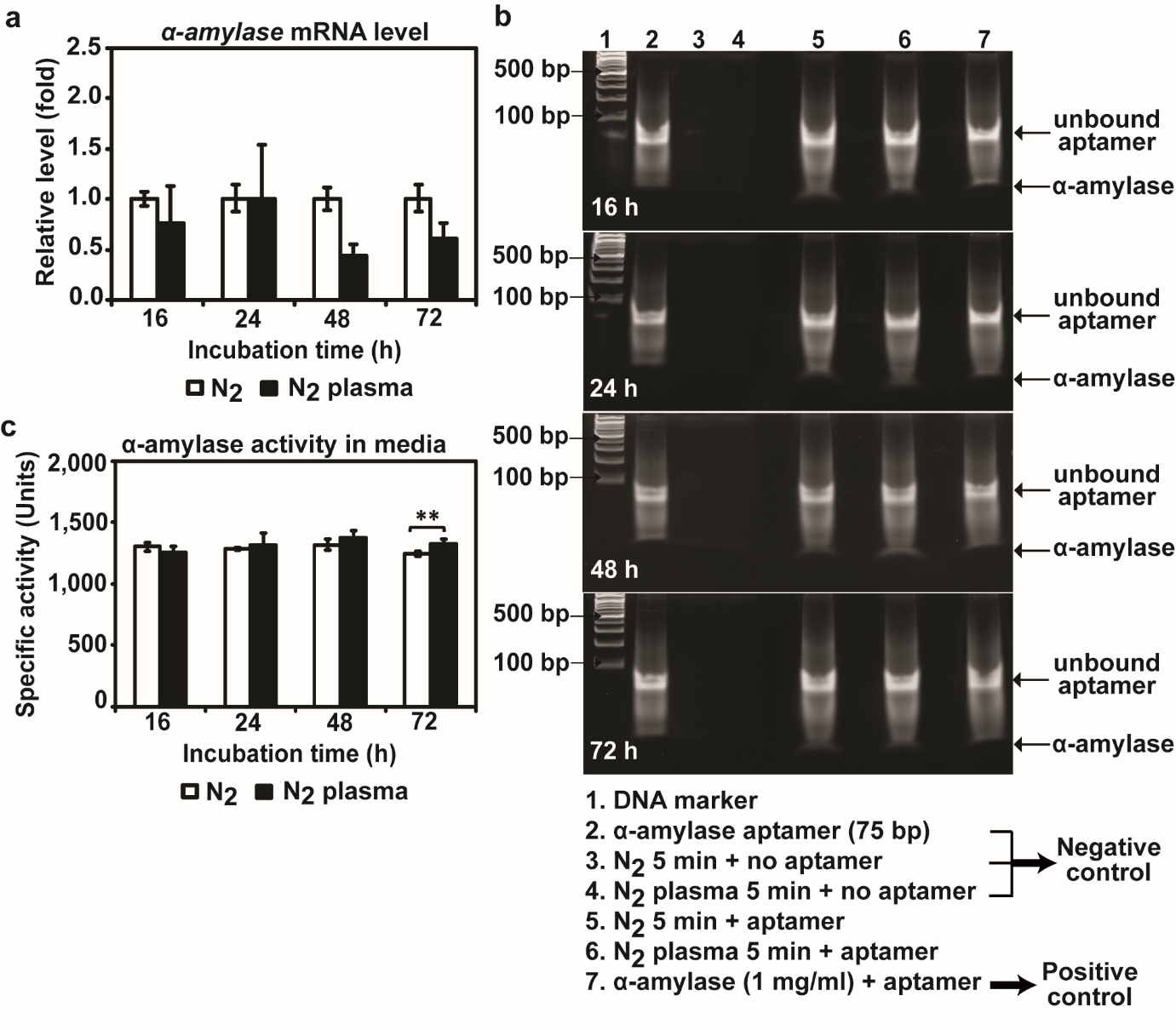
**

**Supplementary Figure S2. The intracellular expression and extracellular secretion of α-amylase in *A. oryzae* hyphae treated with plasma.**

The fungal hyphae grown for 16h was exposed to N_2_ gas (control) or plasma for 5 min and then incubated for 24, 48 and 72 h (see supplementary methods).  **a.** The mRNA level of α-amylase in fungal hyphae quantified using QPCR. **b.** The extracellular α-amylase in PDB detected by α-amylase aptamer in native polyacrylamide gel (8 % in TBE, no SDS) electrophoresis. **c.** The specific α-amylase activity (units) measured in PDB media. In A and C, each value is the mean of 3-9 replicate measurements. ** *p* < 0.01.


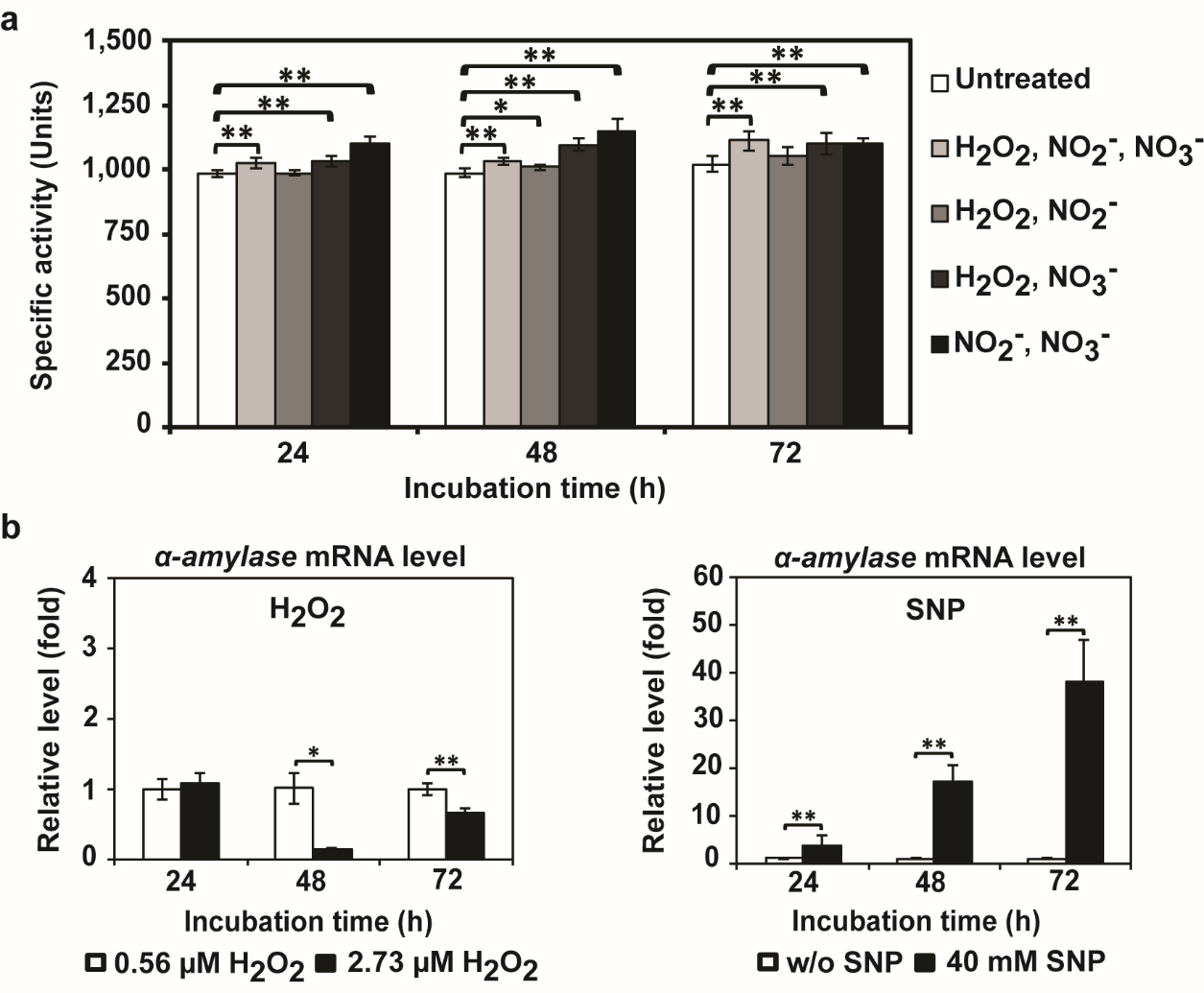


**Supplementary Figure S3. The effect of reactive species on the intracellular expression and extracellular secretion of α-amylase in *A. oryzae*.**

**a.** The specific α-amylase activity (units) measured in PDB media. The fungal spores were incubated in PDB containing the indicated combinations of H_2_O_2_ (2.73 µM), NaNO_2_ (90.49 µM) and NaNO_3_ (14.87 µM) for 24, 48 and 72 h. **b.** The mRNA level of α-amylase in fungal hyphae 24, 48 and 72 h after spores were treated with 0.56 µM (control) H_2_O_2_, 2.73 µM H_2_O_2_ or 40 mM SNP. Each value is the mean of 3-9 replicate measurements. * *p* < 0.05, ** *p* < 0.01.


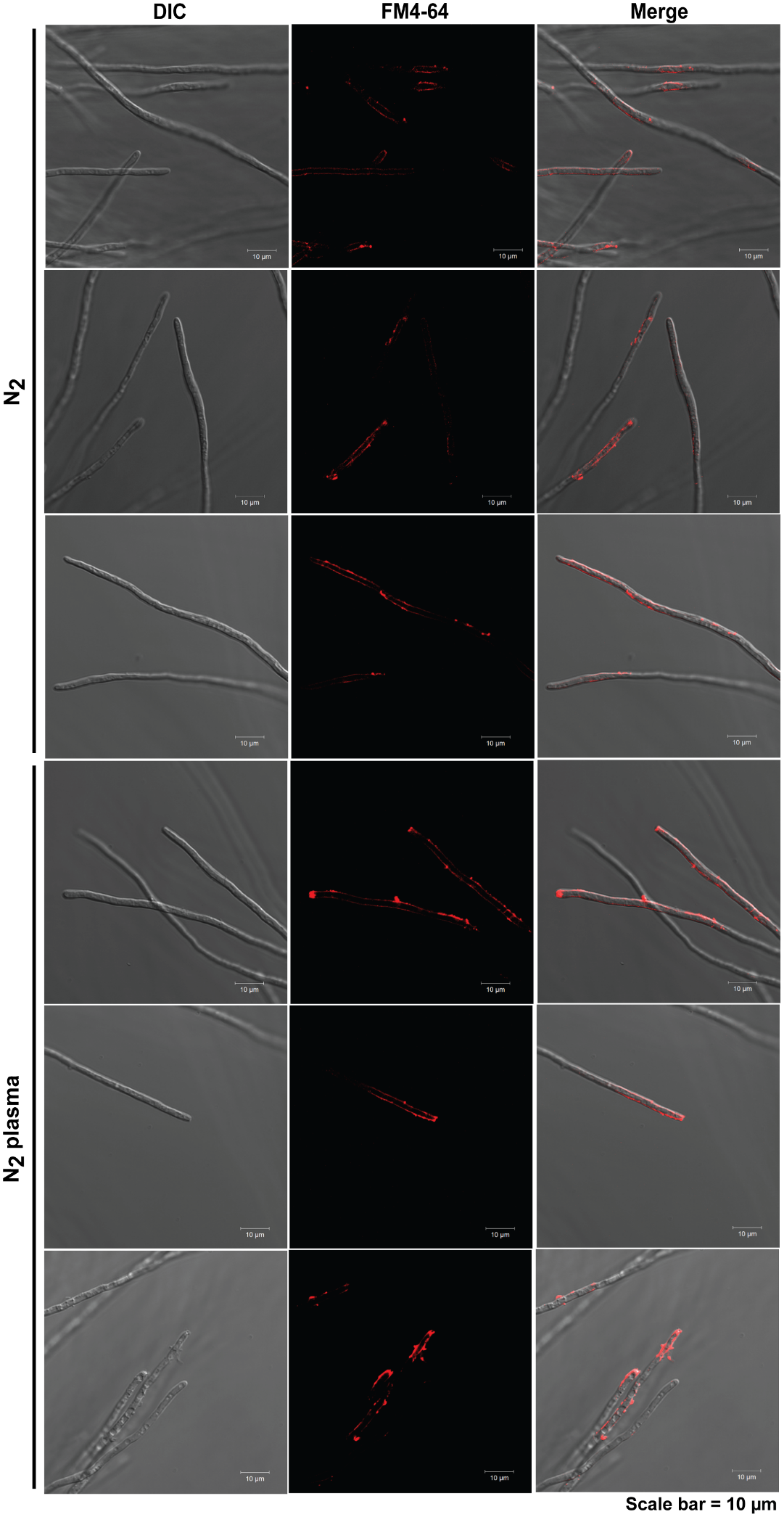


**Supplementary Figure S4**. **Analysis of the vesicles in *A. oryzae* hyphae.**

Secretory protein vesicles in fungal hyphae were stained with FM4-64 (red fluorescence) 24 h after spores were treated with N_2_ gas (control) or plasma for 5 min. Pictures of fungal hypha in 3 different areas were shown. DIC; Differential Interference Contrast, FM4-64; fluorescence, Merge; combined image of DIC and fluorescence.


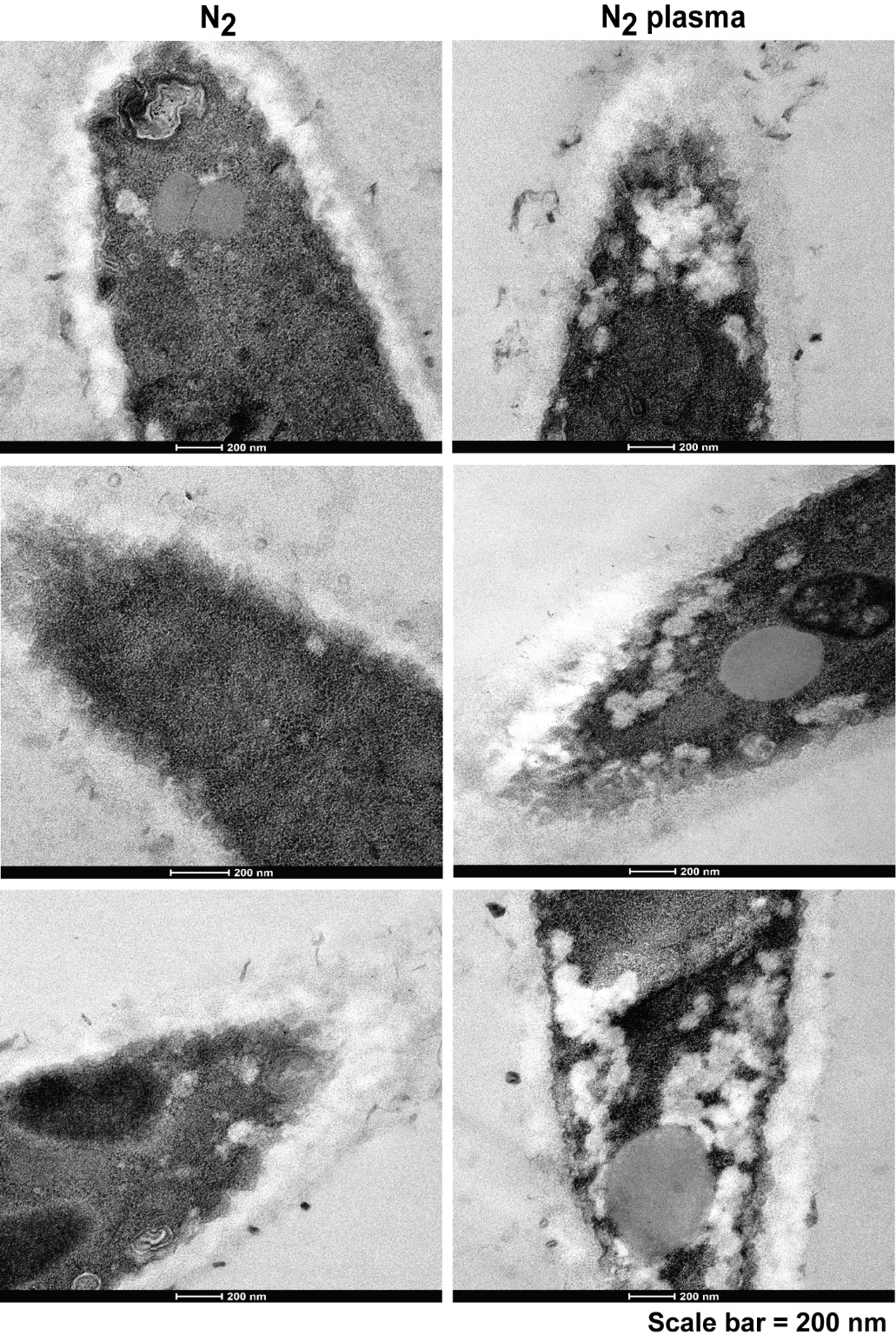


**Supplementary Figure S5. Ultrastructure of fungal hyphae analyzed by TEM.**

Internal structure of fungal hyphal tips was visualized by TEM 24 h after spores were treated with N_2_ gas (control) or plasma for 5 min.


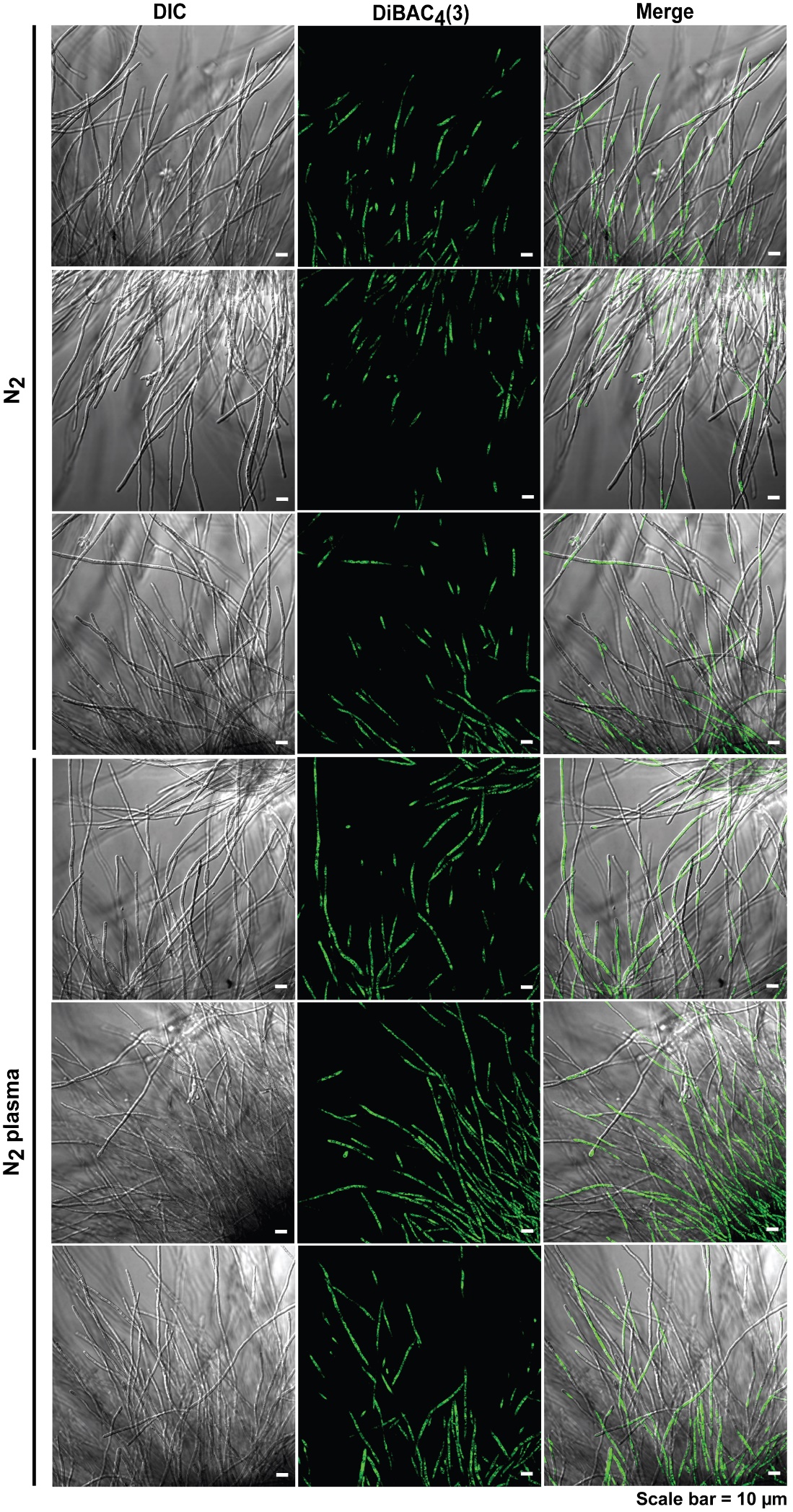


**Supplementary Figure S6**. **Analysis of membrane potential in *A. oryzae* hyphae.**

Membrane depolarization was analyzed in fungal hyphae of 3 different areas using DiBAC_4_(3). Fungal spores were treated with N_2_ gas (control) or plasma for 5 min, and then hyphal membrane depolarization was checked after 24 h. Fluorescence indicates that the cell membrane is depolarized. DIC; Differential Interference Contrast, DiBAC_4_(3); fluorescence, Merge; combined image of DIC and fluorescence

**
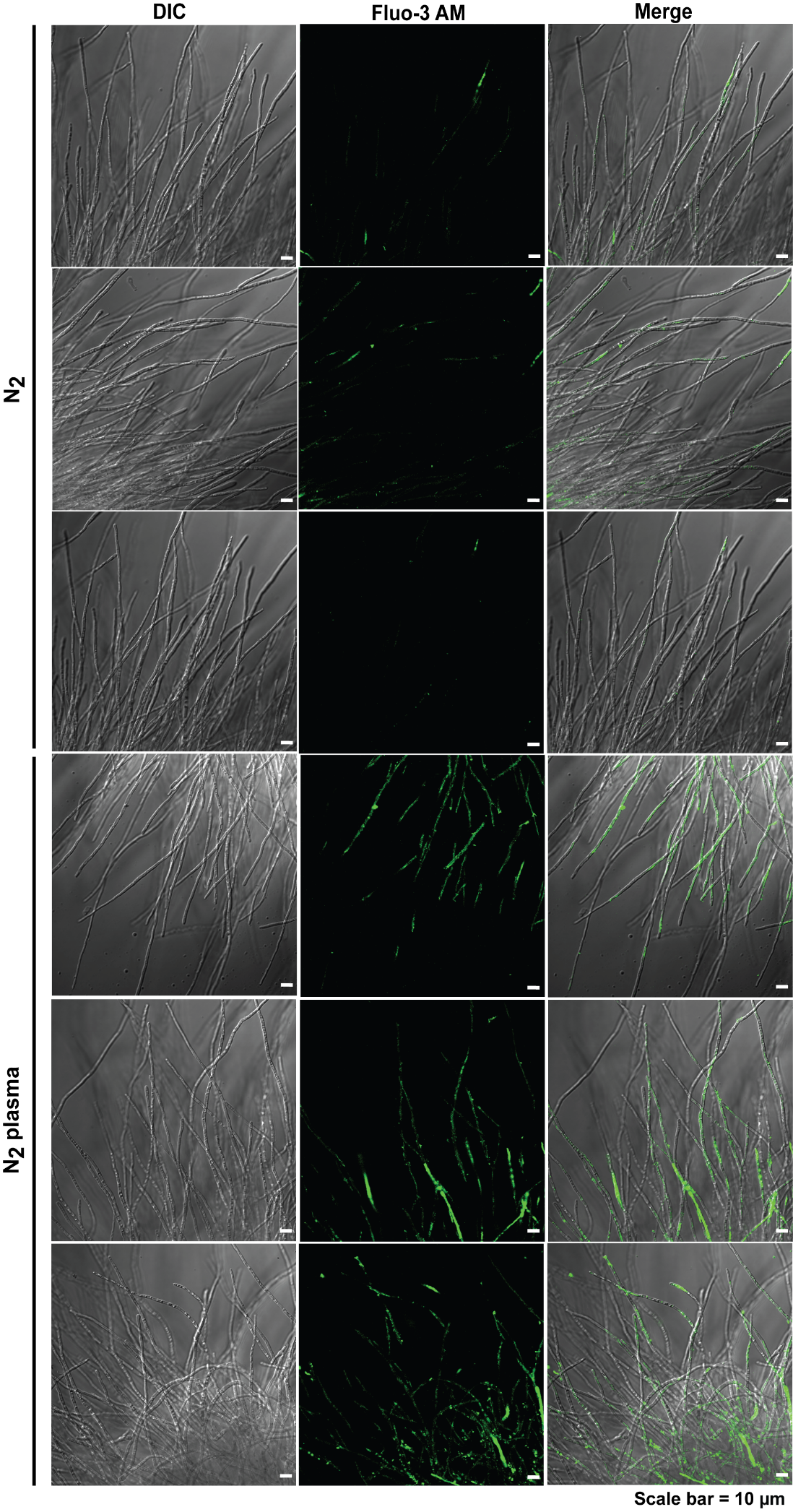
**

**Supplementary Figure S7**. **Intracellular Ca^2+^ level in fungal hyphae.**

Intracellular Ca^2+^ was stained with Fluo-3 AM (green fluorescence) in fungal hyphae grown for 24 h after spores were treated with N_2_ gas (control) or plasma for 5 min. Pictures of fungal hyphae in 3 different areas were shown. DIC; Differential Interference Contrast, Fluo-3 AM; fluorescence, Merge; combined image of DIC and fluorescence

**
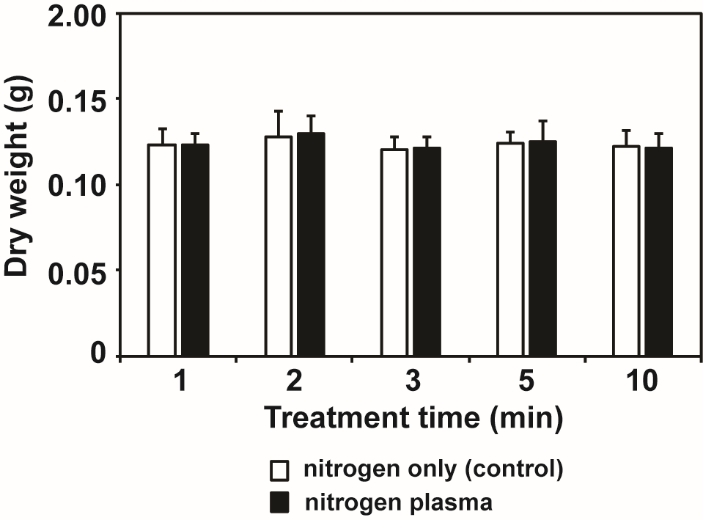
**

**Supplementary Figure S8**. **Total dry weight of fungal mycelia.**

After fungal spores (5 × 10^6^) in PBS were treated with micro DBD nitrogen plasma, spore suspension was transferred into 30 mL PDB media and incubated at 30 °C for 3 days with shaking. Then, fungal mycelia were collected, and dry weight was measured. Each value represents the average of 5 replicate measurements.

**
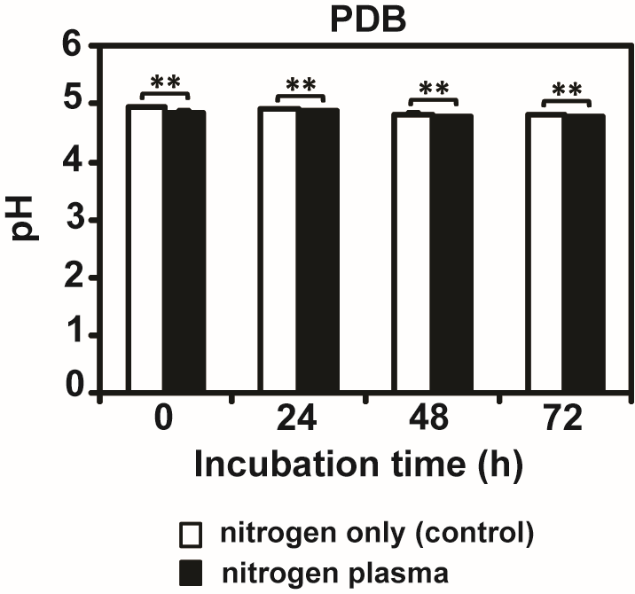
**

**Supplementary Figure S9**.  **pH of PDB incubated for the indicated time after plasma treatment.**

Each value is the average of 6 replicate measurements. ** *p* < 0.01


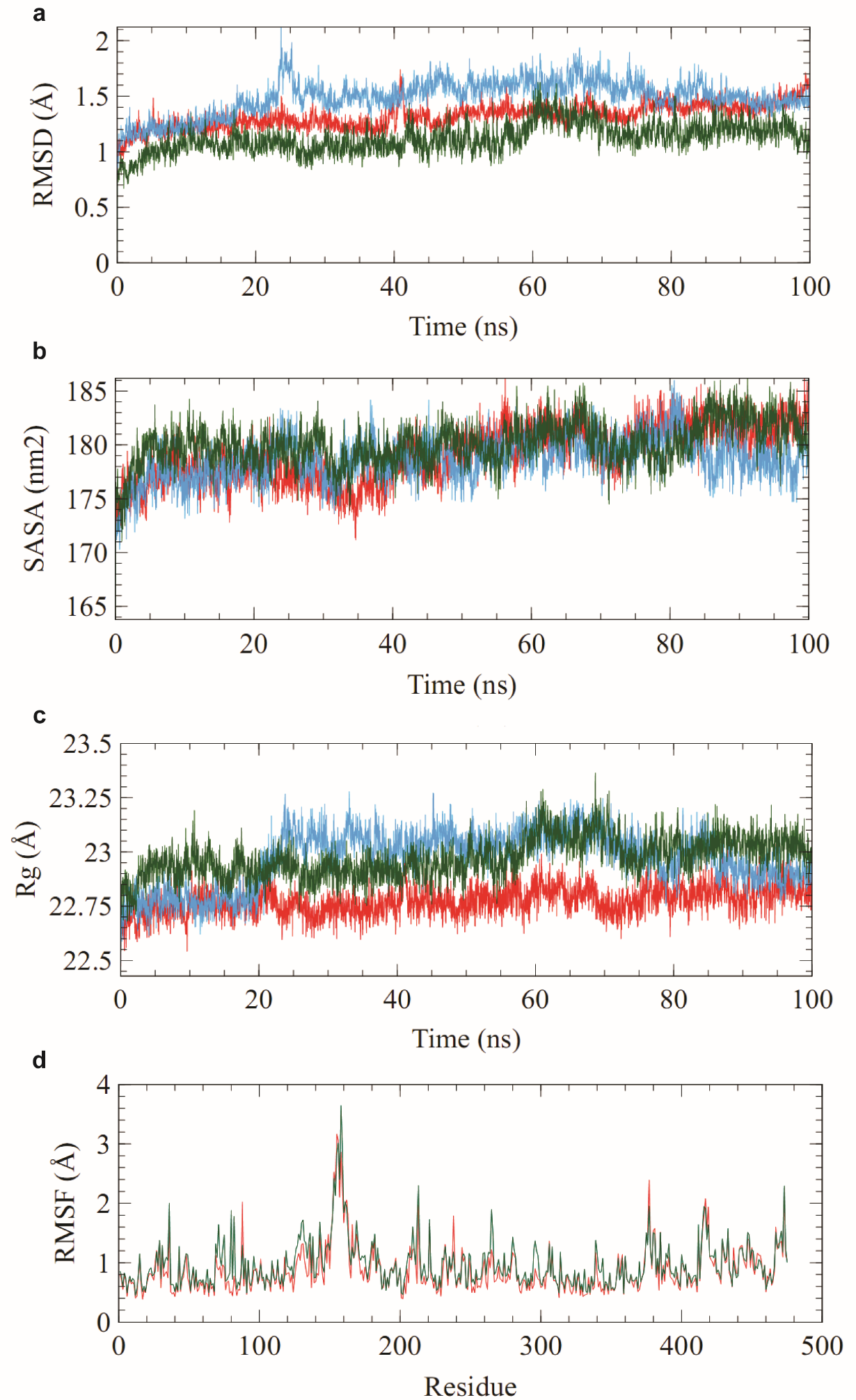


**Supplementary Figure S10**. **Molecular dynamics simulation of α-amylase.**

Changes in protein structural properties of *A. oryzae* α-amylase were examined via simulation analysis under the presence of H_2_O_2_, NO_2_^-^ and NO_3_^-^ at the concentrations measured in PDB and water 0 h and 24 h after plasma treatment (see supplementary methods). Green line represents the α-amylase protein without reactive species while red and blue lines stand for the α-amylase protein with reactive species using concentrations measured in PDB and water 0 h and 24 h after plasma treatment, respectively. **a.** Root-mean-square deviation (RMSD) mainly used for examining protein fluctuation, assesses the variation between the backbones of a protein from its initial structural conformation to final position. **b.** Solvent accessible surface area (SASA) estimates the surface area of protein traced by a solvent molecule. Higher SASA value of a protein represents the relative expansion which is an important factor of the protein stability and folding. **c.** Radius of gyration of protein (Rg) is an indicator of structural compactness of protein. Lower values indicate higher compactness. **d.** Root mean square fluctuation (RMSF) of each residue can be calculated to understand the structural flexibility.

According to the assessment of RSMD and Rg, very slight increase in structural fluctuation and compactness of α-amylase protein was observed under the presence of reactive species. However, the change was observed only during initial time or in 0 h incubation. In addition, SASA and RMSF of α-amylase were not significantly different between samples with and without reactive species. Therefore, the level of reactive species (H_2_O_2_, NO_2_^-^ and NO_3_^-^) generated in PDB after plasma treatment for 5 min may not be enough to cause damage on α-amylase structure, suggesting that α-amylase secreted into PDB may be stable after plasma treatment.

Supplementary Table S1. QPCR analysis for protein secretion related genes

| **Gene** |  | | **Reference gene** | | | **Sample gene** | | |  | | |
| --- | --- | --- | --- | --- | --- | --- | --- | --- | --- | --- | --- |
|  | **Inc. time** | **Treat** | **Ct** | **mean CT** | **mean Calibra** | **Ct** | **mean CT** | **mean Calibre** | **2ddct** | **Mean Flod change** | **SD** |
| **SAR1** | **8 h** | **N_2_** | 11.649 | 11.495 | 11.495 | 23.914 | 23.768 | 23.768 | 1.006 | 1.006 | 0.138 |
|  |  |  | 11.247 |  | 11.495 | 23.723 |  | 23.768 | 0.869 |  |  |
|  |  |  | 11.589 |  | 11.495 | 23.667 |  | 23.768 | 1.144 |  |  |
|  |  | **N_2_ plasma** | 10.594 | 10.653 | 11.495 | 23.614 | 23.534 | 23.768 | 0.596 | 0.658 | 0.072 |
|  |  |  | 10.705 |  | 11.495 | 23.419 |  | 23.768 | 0.737 |  |  |
|  |  |  | 10.660 |  | 11.495 | 23.571 |  | 23.768 | 0.643 |  |  |
|  | **16 h** | **N_2_** | 10.122 | 10.053 | 10.053 | 23.149 | 23.228 | 23.228 | 1.109 | 1.003 | 0.092 |
|  |  |  | 9.910 |  | 10.053 | 23.173 |  | 23.228 | 0.940 |  |  |
|  |  |  | 10.126 |  | 10.053 | 23.362 |  | 23.228 | 0.959 |  |  |
|  |  | **N_2_ plasma** | 10.694 | 10.659 | 10.053 | 23.523 | 23.578 | 23.228 | 1.271 | 1.198 | 0.114 |
|  |  |  | 10.694 |  | 10.053 | 23.539 |  | 23.228 | 1.257 |  |  |
|  |  |  | 10.590 |  | 10.053 | 23.672 |  | 23.228 | 1.067 |  |  |
|  | **24 h** | **N_2_** | 10.542 | 10.548 | 10.548 | 23.893 | 23.933 | 23.933 | 1.023 | 1.003 | 0.095 |
|  |  |  | 10.662 |  | 10.548 | 24.199 |  | 23.933 | 0.900 |  |  |
|  |  |  | 10.440 |  | 10.548 | 23.706 |  | 23.933 | 1.086 |  |  |
|  |  | **N_2_ plasma** | 10.961 | 10.959 | 10.548 | 23.868 | 23.958 | 23.933 | 1.393 | 1.308 | 0.076 |
|  |  |  | 10.958 |  | 10.548 | 23.985 |  | 23.933 | 1.281 |  |  |
|  |  |  | 10.958 |  | 10.548 | 24.022 |  | 23.933 | 1.249 |  |  |
|  | **48 h** | **N_2_** | 10.573 | 10.441 | 10.441 | 24.036 | 23.979 | 23.979 | 1.054 | 1.001 | 0.053 |
|  |  |  | 10.329 |  | 10.441 | 23.866 |  | 23.979 | 1.000 |  |  |
|  |  |  | 10.422 |  | 10.441 | 24.036 |  | 23.979 | 0.949 |  |  |
|  |  | **N_2_ plasma** | 10.500 | 10.498 | 10.441 | 23.757 | 23.769 | 23.979 | 1.215 | 1.204 | 0.018 |
|  |  |  | 10.497 |  | 10.441 | 23.757 |  | 23.979 | 1.213 |  |  |
|  |  |  | 10.497 |  | 10.441 | 23.794 |  | 23.979 | 1.183 |  |  |
|  | **72 h** | **N_2_** | 10.589 | 10.591 | 10.591 | 23.955 | 23.889 | 23.889 | 0.954 | 1.002 | 0.072 |
|  |  |  | 10.510 |  | 10.591 | 23.857 |  | 23.889 | 0.967 |  |  |
|  |  |  | 10.675 |  | 10.591 | 23.857 |  | 23.889 | 1.084 |  |  |
|  |  | **N_2_ plasma** | 11.078 | 11.313 | 10.591 | 24.194 | 24.359 | 23.889 | 1.135 | 1.192 | 0.061 |
|  |  |  | 11.431 |  | 10.591 | 24.483 |  | 23.889 | 1.186 |  |  |
|  |  |  | 11.431 |  | 10.591 | 24.401 |  | 23.889 | 1.255 |  |  |
| **rab2** | **8 h** | **N_2_** | 11.649 | 11.495 | 11.495 | 27.293 | 27.223 | 27.223 | 1.060 | 1.005 | 0.117 |
|  |  |  | 11.247 |  | 11.495 | 27.174 |  | 27.223 | 0.871 |  |  |
|  |  |  | 11.589 |  | 11.495 | 27.201 |  | 27.223 | 1.083 |  |  |
|  |  | **N_2_ plasma** | 10.594 | 10.653 | 11.495 | 27.142 | 27.214 | 27.223 | 0.566 | 0.561 | 0.010 |
|  |  |  | 10.705 |  | 11.495 | 27.250 |  | 27.223 | 0.568 |  |  |
|  |  |  | 10.660 |  | 11.495 | 27.250 |  | 27.223 | 0.550 |  |  |
|  | **16 h** | **N_2_** | 10.122 | 10.053 | 10.053 | 25.450 | 25.448 | 25.448 | 1.048 | 1.002 | 0.085 |
|  |  |  | 9.910 |  | 10.053 | 25.450 |  | 25.448 | 0.904 |  |  |
|  |  |  | 10.126 |  | 10.053 | 25.444 |  | 25.448 | 1.055 |  |  |
|  |  | **N_2_ plasma** | 10.694 | 10.659 | 10.053 | 26.809 | 26.438 | 25.448 | 0.607 | 0.777 | 0.150 |
|  |  |  | 10.694 |  | 10.053 | 26.252 |  | 25.448 | 0.893 |  |  |
|  |  |  | 10.590 |  | 10.053 | 26.252 |  | 25.448 | 0.831 |  |  |
|  | **24 h** | **N_2_** | 10.542 | 10.548 | 10.548 | 26.458 | 26.188 | 26.188 | 0.826 | 1.010 | 0.169 |
|  |  |  | 10.662 |  | 10.548 | 26.088 |  | 26.188 | 1.160 |  |  |
|  |  |  | 10.440 |  | 10.548 | 26.020 |  | 26.188 | 1.043 |  |  |
|  |  | **N_2_ plasma** | 10.961 | 10.959 | 10.548 | 26.807 | 26.617 | 26.188 | 0.867 | 0.992 | 0.112 |
|  |  |  | 10.958 |  | 10.548 | 26.487 |  | 26.188 | 1.081 |  |  |
|  |  |  | 10.958 |  | 10.548 | 26.556 |  | 26.188 | 1.030 |  |  |
|  | **48 h** | **N_2_** | 10.573 | 10.441 | 10.441 | 26.525 | 26.316 | 26.316 | 0.948 | 1.002 | 0.073 |
|  |  |  | 10.329 |  | 10.441 | 26.086 |  | 26.316 | 1.085 |  |  |
|  |  |  | 10.422 |  | 10.441 | 26.337 |  | 26.316 | 0.972 |  |  |
|  |  | **N_2_ plasma** | 10.500 | 10.498 | 10.441 | 26.478 | 26.541 | 26.316 | 0.931 | 0.891 | 0.050 |
|  |  |  | 10.497 |  | 10.441 | 26.512 |  | 26.316 | 0.907 |  |  |
|  |  |  | 10.497 |  | 10.441 | 26.634 |  | 26.316 | 0.834 |  |  |
|  | **72 h** | **N_2_** | 10.589 | 10.591 | 10.591 | 25.482 | 25.503 | 25.503 | 1.012 | 1.000 | 0.037 |
|  |  |  | 10.510 |  | 10.591 | 25.482 |  | 25.503 | 0.959 |  |  |
|  |  |  | 10.675 |  | 10.591 | 25.543 |  | 25.503 | 1.030 |  |  |
|  |  | **N_2_ plasma** | 11.078 | 11.313 | 10.591 | 26.325 | 26.364 | 25.503 | 0.792 | 0.913 | 0.111 |
|  |  |  | 11.431 |  | 10.591 | 26.325 |  | 25.503 | 1.012 |  |  |
|  |  |  | 11.431 |  | 10.591 | 26.441 |  | 25.503 | 0.934 |  |  |
| **YPT1** | **8 h** | **N_2_** | 11.649 | 11.495 | 11.495 | 24.711 | 24.703 | 24.703 | 1.107 | 1.003 | 0.092 |
|  |  |  | 11.247 |  | 11.495 | 24.499 |  | 24.703 | 0.970 |  |  |
|  |  |  | 11.589 |  | 11.495 | 24.900 |  | 24.703 | 0.931 |  |  |
|  |  | **N_2_ plasma** | 10.594 | 10.653 | 11.495 | 24.273 | 24.274 | 24.703 | 0.722 | 0.751 | 0.027 |
|  |  |  | 10.705 |  | 11.495 | 24.281 |  | 24.703 | 0.775 |  |  |
|  |  |  | 10.660 |  | 11.495 | 24.268 |  | 24.703 | 0.758 |  |  |
|  | **16 h** | **N_2_** | 10.122 | 10.053 | 10.053 | 23.844 | 23.823 | 23.823 | 1.035 | 1.002 | 0.070 |
|  |  |  | 9.910 |  | 10.053 | 23.799 |  | 23.823 | 0.921 |  |  |
|  |  |  | 10.126 |  | 10.053 | 23.827 |  | 23.823 | 1.050 |  |  |
|  |  | **N_2_ plasma** | 10.694 | 10.659 | 10.053 | 23.756 | 23.856 | 23.823 | 1.634 | 1.493 | 0.144 |
|  |  |  | 10.694 |  | 10.053 | 23.880 |  | 23.823 | 1.499 |  |  |
|  |  |  | 10.590 |  | 10.053 | 23.932 |  | 23.823 | 1.346 |  |  |
|  | **24 h** | **N_2_** | 10.542 | 10.548 | 10.548 | 24.404 | 24.447 | 24.447 | 1.026 | 1.004 | 0.103 |
|  |  |  | 10.662 |  | 10.548 | 24.432 |  | 24.447 | 1.093 |  |  |
|  |  |  | 10.440 |  | 10.548 | 24.505 |  | 24.447 | 0.892 |  |  |
|  |  | **N_2_ plasma** | 10.961 | 10.959 | 10.548 | 24.010 | 24.210 | 24.447 | 1.803 | 1.577 | 0.215 |
|  |  |  | 10.958 |  | 10.548 | 24.397 |  | 24.447 | 1.375 |  |  |
|  |  |  | 10.958 |  | 10.548 | 24.223 |  | 24.447 | 1.552 |  |  |
|  | **48 h** | **N_2_** | 10.573 | 10.441 | 10.441 | 24.936 | 24.923 | 24.923 | 1.086 | 1.002 | 0.085 |
|  |  |  | 10.329 |  | 10.441 | 24.936 |  | 24.923 | 0.917 |  |  |
|  |  |  | 10.422 |  | 10.441 | 24.897 |  | 24.923 | 1.004 |  |  |
|  |  | **N_2_ plasma** | 10.500 | 10.498 | 10.441 | 24.236 | 24.199 | 24.923 | 1.676 | 1.725 | 0.190 |
|  |  |  | 10.497 |  | 10.441 | 24.027 |  | 24.923 | 1.935 |  |  |
|  |  |  | 10.497 |  | 10.441 | 24.334 |  | 24.923 | 1.563 |  |  |
|  | **72 h** | **N_2_** | 10.589 | 10.591 | 10.591 | 24.388 | 24.319 | 24.319 | 0.951 | 1.001 | 0.046 |
|  |  |  | 10.510 |  | 10.591 | 24.179 |  | 24.319 | 1.042 |  |  |
|  |  |  | 10.675 |  | 10.591 | 24.389 |  | 24.319 | 1.009 |  |  |
|  |  | **N_2_ plasma** | 11.078 | 11.313 | 10.591 | 24.622 | 24.637 | 24.319 | 1.135 | 1.330 | 0.170 |
|  |  |  | 11.431 |  | 10.591 | 24.622 |  | 24.319 | 1.450 |  |  |
|  |  |  | 11.431 |  | 10.591 | 24.667 |  | 24.319 | 1.406 |  |  |
| **SEC4** | **8 h** | **N_2_** | 11.649 | 11.495 | 11.495 | 25.274 | 25.250 | 25.250 | 1.094 | 1.007 | 0.136 |
|  |  |  | 11.247 |  | 11.495 | 25.237 |  | 25.250 | 0.849 |  |  |
|  |  |  | 11.589 |  | 11.495 | 25.238 |  | 25.250 | 1.076 |  |  |
|  |  | **N_2_ plasma** | 10.594 | 10.653 | 11.495 | 24.841 | 25.099 | 25.250 | 0.711 | 0.622 | 0.079 |
|  |  |  | 10.705 |  | 11.495 | 25.296 |  | 25.250 | 0.560 |  |  |
|  |  |  | 10.660 |  | 11.495 | 25.162 |  | 25.250 | 0.596 |  |  |
|  | **16 h** | **N_2_** | 10.122 | 10.053 | 10.053 | 24.158 | 24.176 | 24.176 | 1.063 | 1.010 | 0.170 |
|  |  |  | 9.910 |  | 10.053 | 24.319 |  | 24.176 | 0.820 |  |  |
|  |  |  | 10.126 |  | 10.053 | 24.051 |  | 24.176 | 1.147 |  |  |
|  |  | **N_2_ plasma** | 10.694 | 10.659 | 10.053 | 24.635 | 24.665 | 24.176 | 1.134 | 1.086 | 0.053 |
|  |  |  | 10.694 |  | 10.053 | 24.688 |  | 24.176 | 1.094 |  |  |
|  |  |  | 10.590 |  | 10.053 | 24.672 |  | 24.176 | 1.029 |  |  |
|  | **24 h** | **N_2_** | 10.542 | 10.548 | 10.548 | 24.523 | 24.644 | 24.644 | 1.083 | 1.006 | 0.126 |
|  |  |  | 10.662 |  | 10.548 | 24.655 |  | 24.644 | 1.074 |  |  |
|  |  |  | 10.440 |  | 10.548 | 24.753 |  | 24.644 | 0.860 |  |  |
|  |  | **N_2_ plasma** | 10.961 | 10.959 | 10.548 | 24.829 | 24.881 | 24.644 | 1.171 | 1.132 | 0.109 |
|  |  |  | 10.958 |  | 10.548 | 25.041 |  | 24.644 | 1.009 |  |  |
|  |  |  | 10.958 |  | 10.548 | 24.771 |  | 24.644 | 1.216 |  |  |
|  | **48 h** | **N_2_** | 10.573 | 10.441 | 10.441 | 24.611 | 24.630 | 24.630 | 1.110 | 1.005 | 0.126 |
|  |  |  | 10.329 |  | 10.441 | 24.726 |  | 24.630 | 0.866 |  |  |
|  |  |  | 10.422 |  | 10.441 | 24.553 |  | 24.630 | 1.041 |  |  |
|  |  | **N_2_ plasma** | 10.500 | 10.498 | 10.441 | 24.810 | 24.982 | 24.630 | 0.919 | 0.818 | 0.092 |
|  |  |  | 10.497 |  | 10.441 | 25.122 |  | 24.630 | 0.739 |  |  |
|  |  |  | 10.497 |  | 10.441 | 25.014 |  | 24.630 | 0.797 |  |  |
|  | **72 h** | **N_2_** | 10.589 | 10.591 | 10.591 | 24.528 | 24.565 | 24.565 | 1.024 | 1.001 | 0.051 |
|  |  |  | 10.510 |  | 10.591 | 24.570 |  | 24.565 | 0.942 |  |  |
|  |  |  | 10.675 |  | 10.591 | 24.597 |  | 24.565 | 1.037 |  |  |
|  |  | **N_2_ plasma** | 11.078 | 11.313 | 10.591 | 25.387 | 25.374 | 24.565 | 0.793 | 0.949 | 0.137 |
|  |  |  | 11.431 |  | 10.591 | 25.399 |  | 24.565 | 1.004 |  |  |
|  |  |  | 11.431 |  | 10.591 | 25.335 |  | 24.565 | 1.050 |  |  |
| **rab11** | **8 h** | **N_2_** | 11.649 | 11.495 | 11.495 | 24.347 | 24.404 | 24.404 | 1.158 | 1.011 | 0.179 |
|  |  |  | 11.247 |  | 11.495 | 24.456 |  | 24.404 | 0.812 |  |  |
|  |  |  | 11.589 |  | 11.495 | 24.410 |  | 24.404 | 1.063 |  |  |
|  |  | **N_2_ plasma** | 10.594 | 10.653 | 11.495 | 24.225 | 24.138 | 24.404 | 0.606 | 0.673 | 0.058 |
|  |  |  | 10.705 |  | 11.495 | 24.103 |  | 24.404 | 0.713 |  |  |
|  |  |  | 10.660 |  | 11.495 | 24.085 |  | 24.404 | 0.700 |  |  |
|  | **16 h** | **N_2_** | 10.122 | 10.053 | 10.053 | 23.326 | 23.286 | 23.286 | 1.021 | 1.005 | 0.116 |
|  |  |  | 9.910 |  | 10.053 | 23.326 |  | 23.286 | 0.881 |  |  |
|  |  |  | 10.126 |  | 10.053 | 23.206 |  | 23.286 | 1.112 |  |  |
|  |  | **N_2_ plasma** | 10.694 | 10.659 | 10.053 | 23.831 | 23.889 | 23.286 | 1.069 | 1.004 | 0.057 |
|  |  |  | 10.694 |  | 10.053 | 23.962 |  | 23.286 | 0.976 |  |  |
|  |  |  | 10.590 |  | 10.053 | 23.874 |  | 23.286 | 0.966 |  |  |
|  | **24 h** | **N_2_** | 10.896 | 10.907 | 10.907 | 24.636 | 24.521 | 24.521 | 0.917 | 1.002 | 0.078 |
|  |  |  | 10.912 |  | 10.907 | 24.430 |  | 24.521 | 1.069 |  |  |
|  |  |  | 10.912 |  | 10.907 | 24.496 |  | 24.521 | 1.021 |  |  |
|  |  | **N_2_ plasma** | 11.001 | 10.874 | 10.907 | 24.327 | 24.592 | 24.521 | 1.221 | 0.949 | 0.238 |
|  |  |  | 11.001 |  | 10.907 | 24.852 |  | 24.521 | 0.848 |  |  |
|  |  |  | 10.620 |  | 10.907 | 24.595 |  | 24.521 | 0.779 |  |  |
|  | **48 h** | **N_2_** | 10.573 | 10.441 | 10.441 | 24.265 | 24.241 | 24.241 | 1.077 | 1.002 | 0.085 |
|  |  |  | 10.329 |  | 10.441 | 24.265 |  | 24.241 | 0.909 |  |  |
|  |  |  | 10.422 |  | 10.441 | 24.192 |  | 24.241 | 1.021 |  |  |
|  |  | **N_2_ plasma** | 10.500 | 10.498 | 10.441 | 24.555 | 24.553 | 24.241 | 0.838 | 0.838 | 0.040 |
|  |  |  | 10.497 |  | 10.441 | 24.483 |  | 24.241 | 0.879 |  |  |
|  |  |  | 10.497 |  | 10.441 | 24.622 |  | 24.241 | 0.798 |  |  |
|  | **72 h** | **N_2_** | 10.589 | 10.591 | 10.591 | 24.222 | 24.219 | 24.219 | 0.996 | 1.001 | 0.060 |
|  |  |  | 10.510 |  | 10.591 | 24.222 |  | 24.219 | 0.944 |  |  |
|  |  |  | 10.675 |  | 10.591 | 24.214 |  | 24.219 | 1.064 |  |  |
|  |  | **N_2_ plasma** | 11.078 | 11.313 | 10.591 | 24.935 | 25.177 | 24.219 | 0.853 | 0.852 | 0.086 |
|  |  |  | 11.431 |  | 10.591 | 25.151 |  | 24.219 | 0.938 |  |  |
|  |  |  | 11.431 |  | 10.591 | 25.444 |  | 24.219 | 0.766 |  |  |
| **SEC13** | **8 h** | **N_2_** | 11.649 | 11.495 | 11.495 | 25.100 | 24.891 | 24.891 | 0.963 | 1.000 | 0.033 |
|  |  |  | 11.247 |  | 11.495 | 24.625 |  | 24.891 | 1.013 |  |  |
|  |  |  | 11.589 |  | 11.495 | 24.948 |  | 24.891 | 1.026 |  |  |
|  |  | **N_2_ plasma** | 10.594 | 10.653 | 11.495 | 24.654 | 24.537 | 24.891 | 0.631 | 0.716 | 0.082 |
|  |  |  | 10.705 |  | 11.495 | 24.434 |  | 24.891 | 0.794 |  |  |
|  |  |  | 10.660 |  | 11.495 | 24.523 |  | 24.891 | 0.723 |  |  |
|  | **16 h** | **N_2_** | 10.122 | 10.053 | 10.053 | 24.297 | 24.281 | 24.281 | 1.038 | 1.003 | 0.095 |
|  |  |  | 9.910 |  | 10.053 | 24.297 |  | 24.281 | 0.896 |  |  |
|  |  |  | 10.126 |  | 10.053 | 24.251 |  | 24.281 | 1.075 |  |  |
|  |  | **N_2_ plasma** | 10.694 | 10.659 | 10.053 | 24.867 | 24.784 | 24.281 | 1.039 | 1.075 | 0.031 |
|  |  |  | 10.694 |  | 10.053 | 24.789 |  | 24.281 | 1.097 |  |  |
|  |  |  | 10.590 |  | 10.053 | 24.696 |  | 24.281 | 1.089 |  |  |
|  | **24 h** | **N_2_** | 10.896 | 10.907 | 10.907 | 25.737 | 25.446 | 25.446 | 0.812 | 1.011 | 0.176 |
|  |  |  | 10.912 |  | 10.907 | 25.257 |  | 25.446 | 1.144 |  |  |
|  |  |  | 10.912 |  | 10.907 | 25.345 |  | 25.446 | 1.077 |  |  |
|  |  | **N_2_ plasma** | 11.001 | 10.874 | 10.907 | 25.406 | 25.535 | 25.446 | 1.098 | 0.938 | 0.217 |
|  |  |  | 11.001 |  | 10.907 | 25.505 |  | 25.446 | 1.025 |  |  |
|  |  |  | 10.620 |  | 10.907 | 25.695 |  | 25.446 | 0.690 |  |  |
|  | **48 h** | **N_2_** | 10.573 | 10.441 | 10.441 | 25.522 | 25.457 | 25.457 | 1.048 | 1.004 | 0.105 |
|  |  |  | 10.329 |  | 10.441 | 25.522 |  | 25.457 | 0.884 |  |  |
|  |  |  | 10.422 |  | 10.441 | 25.327 |  | 25.457 | 1.080 |  |  |
|  |  | **N_2_ plasma** | 10.500 | 10.498 | 10.441 | 25.854 | 25.778 | 25.457 | 0.791 | 0.835 | 0.078 |
|  |  |  | 10.497 |  | 10.441 | 25.854 |  | 25.457 | 0.789 |  |  |
|  |  |  | 10.497 |  | 10.441 | 25.625 |  | 25.457 | 0.925 |  |  |
|  | **72 h** | **N_2_** | 10.589 | 10.591 | 10.591 | 25.101 | 25.113 | 25.113 | 1.007 | 1.001 | 0.045 |
|  |  |  | 10.510 |  | 10.591 | 25.101 |  | 25.113 | 0.953 |  |  |
|  |  |  | 10.675 |  | 10.591 | 25.137 |  | 25.113 | 1.042 |  |  |
|  |  | **N_2_ plasma** | 11.078 | 11.313 | 10.591 | 25.934 | 25.949 | 25.113 | 0.793 | 0.929 | 0.120 |
|  |  |  | 11.431 |  | 10.591 | 25.928 |  | 25.113 | 1.018 |  |  |
|  |  |  | 11.431 |  | 10.591 | 25.986 |  | 25.113 | 0.977 |  |  |
| **INO1** | **8 h** | **N_2_** | 11.649 | 11.495 | 11.495 | 25.296 | 25.438 | 25.438 | 1.227 | 1.011 | 0.189 |
|  |  |  | 11.247 |  | 11.495 | 25.296 |  | 25.438 | 0.929 |  |  |
|  |  |  | 11.589 |  | 11.495 | 25.720 |  | 25.438 | 0.877 |  |  |
|  |  | **N_2_ plasma** | 10.594 | 10.653 | 11.495 | 25.107 | 25.160 | 25.438 | 0.673 | 0.677 | 0.038 |
|  |  |  | 10.705 |  | 11.495 | 25.127 |  | 25.438 | 0.717 |  |  |
|  |  |  | 10.660 |  | 11.495 | 25.244 |  | 25.438 | 0.641 |  |  |
|  | **16 h** | **N_2_** | 10.122 | 10.053 | 10.053 | 24.339 | 24.556 | 24.556 | 1.220 | 1.012 | 0.193 |
|  |  |  | 9.910 |  | 10.053 | 24.664 |  | 24.556 | 0.840 |  |  |
|  |  |  | 10.126 |  | 10.053 | 24.664 |  | 24.556 | 0.976 |  |  |
|  |  | **N_2_ plasma** | 10.694 | 10.659 | 10.053 | 24.686 | 24.728 | 24.556 | 1.425 | 1.355 | 0.121 |
|  |  |  | 10.694 |  | 10.053 | 24.686 |  | 24.556 | 1.425 |  |  |
|  |  |  | 10.590 |  | 10.053 | 24.813 |  | 24.556 | 1.215 |  |  |
|  | **24 h** | **N_2_** | 10.896 | 10.907 | 10.907 | 26.125 | 26.136 | 26.136 | 1.000 | 1.000 | 0.017 |
|  |  |  | 10.912 |  | 10.907 | 26.117 |  | 26.136 | 1.017 |  |  |
|  |  |  | 10.912 |  | 10.907 | 26.166 |  | 26.136 | 0.983 |  |  |
|  |  | **N_2_ plasma** | 11.001 | 10.874 | 10.907 | 26.208 | 26.211 | 26.136 | 1.015 | 0.934 | 0.128 |
|  |  |  | 11.001 |  | 10.907 | 26.229 |  | 26.136 | 1.000 |  |  |
|  |  |  | 10.620 |  | 10.907 | 26.195 |  | 26.136 | 0.787 |  |  |
|  | **48 h** | **N_2_** | 10.573 | 10.441 | 10.441 | 26.632 | 26.556 | 26.556 | 1.039 | 1.000 | 0.035 |
|  |  |  | 10.329 |  | 10.441 | 26.485 |  | 26.556 | 0.971 |  |  |
|  |  |  | 10.422 |  | 10.441 | 26.550 |  | 26.556 | 0.991 |  |  |
|  |  | **N_2_ plasma** | 10.500 | 10.498 | 10.441 | 26.422 | 26.525 | 26.556 | 1.143 | 1.065 | 0.078 |
|  |  |  | 10.497 |  | 10.441 | 26.632 |  | 26.556 | 0.986 |  |  |
|  |  |  | 10.497 |  | 10.441 | 26.520 |  | 26.556 | 1.066 |  |  |
|  | **72 h** | **N_2_** | 10.589 | 10.591 | 10.591 | 27.180 | 27.151 | 27.151 | 0.978 | 1.002 | 0.068 |
|  |  |  | 10.510 |  | 10.591 | 26.961 |  | 27.151 | 1.078 |  |  |
|  |  |  | 10.675 |  | 10.591 | 27.312 |  | 27.151 | 0.948 |  |  |
|  |  | **N_2_ plasma** | 11.078 | 11.313 | 10.591 | 28.086 | 28.006 | 27.151 | 0.733 | 0.923 | 0.164 |
|  |  |  | 11.431 |  | 10.591 | 27.973 |  | 27.151 | 1.012 |  |  |
|  |  |  | 11.431 |  | 10.591 | 27.959 |  | 27.151 | 1.023 |  |  |
| **SEC14** | **8 h** | **N_2_** | 11.649 | 11.495 | 11.495 | 25.184 | 25.051 | 25.051 | 1.015 | 1.022 | 0.258 |
|  |  |  | 11.247 |  | 11.495 | 25.184 |  | 25.051 | 0.768 |  |  |
|  |  |  | 11.589 |  | 11.495 | 24.785 |  | 25.051 | 1.284 |  |  |
|  |  | **N_2_ plasma** | 10.594 | 10.653 | 11.495 | 24.647 | 24.604 | 25.051 | 0.708 | 0.762 | 0.052 |
|  |  |  | 10.705 |  | 11.495 | 24.648 |  | 25.051 | 0.765 |  |  |
|  |  |  | 10.660 |  | 11.495 | 24.515 |  | 25.051 | 0.813 |  |  |
|  | **16 h** | **N_2_** | 10.122 | 10.053 | 10.053 | 24.220 | 24.212 | 24.212 | 1.044 | 1.001 | 0.057 |
|  |  |  | 9.910 |  | 10.053 | 24.164 |  | 24.212 | 0.936 |  |  |
|  |  |  | 10.126 |  | 10.053 | 24.253 |  | 24.212 | 1.023 |  |  |
|  |  | **N_2_ plasma** | 10.694 | 10.659 | 10.053 | 24.699 | 24.698 | 24.212 | 1.113 | 1.088 | 0.043 |
|  |  |  | 10.694 |  | 10.053 | 24.699 |  | 24.212 | 1.113 |  |  |
|  |  |  | 10.590 |  | 10.053 | 24.696 |  | 24.212 | 1.038 |  |  |
|  | **24 h** | **N_2_** | 10.896 | 10.907 | 10.907 | 25.445 | 25.518 | 25.518 | 1.045 | 1.001 | 0.050 |
|  |  |  | 10.912 |  | 10.907 | 25.507 |  | 25.518 | 1.012 |  |  |
|  |  |  | 10.912 |  | 10.907 | 25.603 |  | 25.518 | 0.946 |  |  |
|  |  | **N_2_ plasma** | 11.001 | 10.874 | 10.907 | 25.504 | 25.523 | 25.518 | 1.078 | 0.984 | 0.163 |
|  |  |  | 11.001 |  | 10.907 | 25.504 |  | 25.518 | 1.078 |  |  |
|  |  |  | 10.620 |  | 10.907 | 25.561 |  | 25.518 | 0.796 |  |  |
|  | **48 h** | **N_2_** | 10.573 | 10.441 | 10.441 | 25.683 | 25.625 | 25.625 | 1.053 | 1.003 | 0.100 |
|  |  |  | 10.329 |  | 10.441 | 25.683 |  | 25.625 | 0.889 |  |  |
|  |  |  | 10.422 |  | 10.441 | 25.509 |  | 25.625 | 1.069 |  |  |
|  |  | **N_2_ plasma** | 10.500 | 10.498 | 10.441 | 25.912 | 26.020 | 25.625 | 0.854 | 0.792 | 0.053 |
|  |  |  | 10.497 |  | 10.441 | 26.074 |  | 25.625 | 0.762 |  |  |
|  |  |  | 10.497 |  | 10.441 | 26.074 |  | 25.625 | 0.762 |  |  |
|  | **72 h** | **N_2_** | 10.589 | 10.591 | 10.591 | 25.985 | 26.042 | 26.042 | 1.039 | 1.005 | 0.120 |
|  |  |  | 10.510 |  | 10.591 | 26.158 |  | 26.042 | 0.872 |  |  |
|  |  |  | 10.675 |  | 10.591 | 25.983 |  | 26.042 | 1.104 |  |  |
|  |  | **N_2_ plasma** | 11.078 | 11.313 | 10.591 | 26.841 | 26.972 | 26.042 | 0.805 | 0.868 | 0.074 |
|  |  |  | 11.431 |  | 10.591 | 26.957 |  | 26.042 | 0.949 |  |  |
|  |  |  | 11.431 |  | 10.591 | 27.116 |  | 26.042 | 0.850 |  |  |
| **SEC18** | **8 h** | **N_2_** | 11.649 | 11.495 | 11.495 | 26.083 | 26.095 | 26.095 | 1.123 | 1.007 | 0.141 |
|  |  |  | 11.247 |  | 11.495 | 26.083 |  | 26.095 | 0.849 |  |  |
|  |  |  | 11.589 |  | 11.495 | 26.120 |  | 26.095 | 1.049 |  |  |
|  |  | **N_2_ plasma** | 10.594 | 10.653 | 11.495 | 26.049 | 25.877 | 26.095 | 0.553 | 0.656 | 0.121 |
|  |  |  | 10.705 |  | 11.495 | 25.646 |  | 26.095 | 0.790 |  |  |
|  |  |  | 10.660 |  | 11.495 | 25.937 |  | 26.095 | 0.626 |  |  |
|  | **16 h** | **N_2_** | 10.122 | 10.053 | 10.053 | 25.577 | 25.389 | 25.389 | 0.921 | 1.005 | 0.124 |
|  |  |  | 9.910 |  | 10.053 | 25.325 |  | 25.389 | 0.946 |  |  |
|  |  |  | 10.126 |  | 10.053 | 25.265 |  | 25.389 | 1.147 |  |  |
|  |  | **N_2_ plasma** | 10.694 | 10.659 | 10.053 | 26.073 | 26.298 | 25.389 | 0.971 | 0.818 | 0.134 |
|  |  |  | 10.694 |  | 10.053 | 26.430 |  | 25.389 | 0.758 |  |  |
|  |  |  | 10.590 |  | 10.053 | 26.391 |  | 25.389 | 0.725 |  |  |
|  | **24 h** | **N_2_** | 10.896 | 10.907 | 10.907 | 26.166 | 26.207 | 26.207 | 1.021 | 1.000 | 0.027 |
|  |  |  | 10.912 |  | 10.907 | 26.198 |  | 26.207 | 1.010 |  |  |
|  |  |  | 10.912 |  | 10.907 | 26.256 |  | 26.207 | 0.970 |  |  |
|  |  | **N_2_ plasma** | 11.001 | 10.874 | 10.907 | 26.400 | 26.372 | 26.207 | 0.934 | 0.875 | 0.082 |
|  |  |  | 11.001 |  | 10.907 | 26.438 |  | 26.207 | 0.909 |  |  |
|  |  |  | 10.620 |  | 10.907 | 26.278 |  | 26.207 | 0.781 |  |  |
|  | **48 h** | **N_2_** | 10.573 | 10.441 | 10.441 | 26.401 | 26.183 | 26.183 | 0.942 | 1.005 | 0.127 |
|  |  |  | 10.329 |  | 10.441 | 26.187 |  | 26.183 | 0.922 |  |  |
|  |  |  | 10.422 |  | 10.441 | 25.961 |  | 26.183 | 1.151 |  |  |
|  |  | **N_2_ plasma** | 10.500 | 10.498 | 10.441 | 26.153 | 26.203 | 26.183 | 1.063 | 1.026 | 0.032 |
|  |  |  | 10.497 |  | 10.441 | 26.227 |  | 26.183 | 1.009 |  |  |
|  |  |  | 10.497 |  | 10.441 | 26.228 |  | 26.183 | 1.008 |  |  |
|  | **72 h** | **N_2_** | 10.589 | 10.591 | 10.591 | 26.561 | 26.520 | 26.520 | 0.970 | 1.001 | 0.046 |
|  |  |  | 10.510 |  | 10.591 | 26.470 |  | 26.520 | 0.978 |  |  |
|  |  |  | 10.675 |  | 10.591 | 26.528 |  | 26.520 | 1.054 |  |  |
|  |  | **N_2_ plasma** | 11.078 | 11.313 | 10.591 | 27.192 | 27.208 | 26.520 | 0.879 | 1.032 | 0.159 |
|  |  |  | 11.431 |  | 10.591 | 27.101 |  | 26.520 | 1.197 |  |  |
|  |  |  | 11.431 |  | 10.591 | 27.331 |  | 26.520 | 1.020 |  |  |
| **PMR1** | **8 h** | **N_2_** | 11.649 | 11.495 | 11.495 | 29.902 | 30.053 | 30.053 | 1.236 | 1.012 | 0.199 |
|  |  |  | 11.247 |  | 11.495 | 29.885 |  | 30.053 | 0.946 |  |  |
|  |  |  | 11.589 |  | 11.495 | 30.372 |  | 30.053 | 0.855 |  |  |
|  |  | **N_2_ plasma** | 10.594 | 10.653 | 11.495 | 29.426 | 29.386 | 30.053 | 0.827 | 0.888 | 0.089 |
|  |  |  | 10.705 |  | 11.495 | 29.277 |  | 30.053 | 0.991 |  |  |
|  |  |  | 10.660 |  | 11.495 | 29.456 |  | 30.053 | 0.848 |  |  |
|  | **16 h** | **N_2_** | 10.122 | 10.053 | 10.053 | 28.172 | 27.856 | 27.856 | 0.843 | 1.009 | 0.166 |
|  |  |  | 9.910 |  | 10.053 | 27.697 |  | 27.856 | 1.011 |  |  |
|  |  |  | 10.126 |  | 10.053 | 27.697 |  | 27.856 | 1.174 |  |  |
|  |  | **N_2_ plasma** | 10.694 | 10.659 | 10.053 | 28.607 | 28.832 | 27.856 | 0.927 | 0.798 | 0.223 |
|  |  |  | 10.694 |  | 10.053 | 28.607 |  | 27.856 | 0.927 |  |  |
|  |  |  | 10.590 |  | 10.053 | 29.283 |  | 27.856 | 0.540 |  |  |
|  | **24 h** | **N_2_** | 10.896 | 10.907 | 10.907 | 28.947 | 28.908 | 28.908 | 0.966 | 1.002 | 0.073 |
|  |  |  | 10.912 |  | 10.907 | 28.795 |  | 28.908 | 1.085 |  |  |
|  |  |  | 10.912 |  | 10.907 | 28.982 |  | 28.908 | 0.953 |  |  |
|  |  | **N_2_ plasma** | 11.001 | 10.874 | 10.907 | 29.144 | 29.114 | 28.908 | 0.907 | 0.853 | 0.118 |
|  |  |  | 11.001 |  | 10.907 | 29.099 |  | 28.908 | 0.935 |  |  |
|  |  |  | 10.620 |  | 10.907 | 29.099 |  | 28.908 | 0.718 |  |  |
|  | **48 h** | **N_2_** | 10.573 | 10.441 | 10.441 | 28.982 | 28.777 | 28.777 | 0.951 | 1.003 | 0.099 |
|  |  |  | 10.329 |  | 10.441 | 28.751 |  | 28.777 | 0.942 |  |  |
|  |  |  | 10.422 |  | 10.441 | 28.599 |  | 28.777 | 1.117 |  |  |
|  |  | **N_2_ plasma** | 10.500 | 10.498 | 10.441 | 29.057 | 29.107 | 28.777 | 0.858 | 0.830 | 0.066 |
|  |  |  | 10.497 |  | 10.441 | 29.242 |  | 28.777 | 0.754 |  |  |
|  |  |  | 10.497 |  | 10.441 | 29.023 |  | 28.777 | 0.877 |  |  |
|  | **72 h** | **N_2_** | 10.589 | 10.591 | 10.591 | 28.414 | 28.546 | 28.546 | 1.094 | 1.007 | 0.142 |
|  |  |  | 10.510 |  | 10.591 | 28.711 |  | 28.546 | 0.843 |  |  |
|  |  |  | 10.675 |  | 10.591 | 28.513 |  | 28.546 | 1.084 |  |  |
|  |  | **N_2_ plasma** | 11.078 | 11.313 | 10.591 | 29.118 | 29.162 | 28.546 | 0.943 | 1.082 | 0.132 |
|  |  |  | 11.431 |  | 10.591 | 29.118 |  | 28.546 | 1.204 |  |  |
|  |  |  | 11.431 |  | 10.591 | 29.250 |  | 28.546 | 1.099 |  |  |

**Supplementary References**

Hosen, S. M. Z., Dash, R., Junaid, M., Mitra, S., and Absar, N. (2019) Identification and structural characterization of deleterious non-synonymous single nucleotide polymorphisms in the human SKP2 gene. *Comput Biol Chem* **79:** 127-136.
